# Amino acid homeostasis is a target of metformin therapy

**DOI:** 10.1016/j.molmet.2023.101750

**Published:** 2023-06-09

**Authors:** Calum Forteath, Ify Mordi, Raid Nisr, Erika J. Gutierrez-Lara, Noor Alqurashi, Iain R. Phair, Amy R. Cameron, Craig Beall, Ibrahim Bahr, Mohapradeep Mohan, Aaron K.F. Wong, Adel Dihoum, Anwar Mohammad, Colin N.A. Palmer, Douglas Lamont, Kei Sakamoto, Benoit Viollet, Marc Foretz, Chim C. Lang, Graham Rena

**Affiliations:** 1 Division of Cellular and Systems Medicine, Ninewells Hospital and Medical School, University of Dundee, Dundee, Scotland, DD1 9SY, UK; 2 Division of Molecular and Clinical Medicine, Ninewells Hospital and Medical School, University of Dundee, Dundee, Scotland, DD1 9SY, UK; 3 Department of Clinical and Biomedical Sciences, Faculty of Health and Life Sciences, University of Exeter, RILD Building, Exeter, EX2 5DW, UK; 4 Public Health and Epidemiology Department, Dasman Diabetes Institute, Kuwait City, Kuwait; 5 Division of Population Health and Genomics, Ninewells Hospital and Medical School, University of Dundee, Dundee, Scotland, DD1 9SY, UK; 6 Centre for Advanced Scientific Technologies, School of Life Sciences, University of Dundee, Dundee, DD1 5EH, UK; 7 Novo Nordisk Foundation Center for Basic Metabolic Research, University of Copenhagen, Copenhagen, 2200, Denmark; 8 Université Paris Cité, CNRS, Inserm, Institut Cochin, Paris, 75014, France

**Keywords:** Metformin, Branched chain amino acids, mTOR, SNAT2, Glutamine, Rapamycin, AMPK

## Abstract

**Objective:**

Unexplained changes in regulation of branched chain amino acids (BCAA) during diabetes therapy with metformin have been known for years. Here we have investigated mechanisms underlying this effect.

**Methods:**

We used cellular approaches, including single gene/protein measurements, as well as systems-level proteomics. Findings were then cross-validated with electronic health records and other data from human material.

**Results:**

In cell studies, we observed diminished uptake/incorporation of amino acids following metformin treatment of liver cells and cardiac myocytes. Supplementation of media with amino acids attenuated known effects of the drug, including on glucose production, providing a possible explanation for discrepancies between effective doses *in vivo* and *in vitro* observed in most studies. Data-Independent Acquisition proteomics identified that SNAT2, which mediates tertiary control of BCAA uptake, was the most strongly suppressed amino acid transporter in liver cells following metformin treatment. Other transporters were affected to a lesser extent. In humans, metformin attenuated increased risk of left ventricular hypertrophy due to the AA allele of KLF15, which is an inducer of BCAA catabolism. In plasma from a double-blind placebo-controlled trial in nondiabetic heart failure (trial registration: NCT00473876), metformin caused selective accumulation of plasma BCAA and glutamine, consistent with the effects in cells.

**Conclusions:**

Metformin restricts tertiary control of BCAA cellular uptake. We conclude that modulation of amino acid homeostasis contributes to therapeutic actions of the drug.

## Highlights

• A mechanism for metformin on left ventricular hypertrophy validated in humans.
• An explanation for discrepancies between effective metformin dose *in vivo* and *in vitro*.
• A mechanism for metformin regulating the mTOR amino acid signalling pathway.

## 1 Introduction

Metformin is a key drug in treatment of type 2 diabetes mellitus (DM) [1,2]. Compared with other DM treatments, metformin monotherapy is associated with fewer adverse cardiovascular events, in both clinical trials and in observational studies [[3], [4], [5]]. The reasons for this relative benefit are unclear and the molecular actions of metformin have only recently come to be better defined. The main clinical hallmark of metformin in DM therapy is suppression of gluconeogenesis [2,[6], [7], [8], [9]] and the most likely cellular effect underlying this response is a modest inhibition of mitochondrial complex I in the electron transport chain, while other mechanisms such as modulation of redox metabolism or presenilin enhancer 2 (PEN2) have also been proposed [[10], [11], [12], [13]]. Recently we presented evidence suggesting the effect of metformin on complex I is to reduce efficiency of coupling of NADH oxidation with proton transfer [14]. Compelling evidence for an allosteric effect of AMP on fructose 1,6 bisphosphatase-1 (FBP1), demonstrated that this mediates acute suppression of hepatic glucose production (HGP) by metformin; however, that longer term effects also involve other mechanisms [15]. In clinical studies, metformin is known to acutely raise concentrations of branched chain amino acids (BCAA) in humans [16] but because raised BCAA also associates with overweight [17,18], insulin resistance [18,19] and diabetes [20,21], there has not been much investigation of the possibility that metformin could act therapeutically through effects on amino acids.

Understanding of regulation of amino acids by metformin could however potentially resolve more than one of the remaining uncertainties concerning metformin. Firstly, involvement of BCAA in metformin action might account for some of the discrepancy between effective doses of drug *in vitro* and in plasma *in vivo*, noted by most but not all investigators, as amino acids are commonly present in concentrations at least five-fold higher in cell culture medium than in plasma [22]. Indeed, the highest concentrations of metformin observed *in vivo* are in the gut and there is evidence that this might be a major locus of action of the drug [23,24].

Secondly, regulation of BCAA could clarify the effect of metformin on hepatocyte glucose production. Evidence has been gathering for decades that glucagon, besides being a counterregulatory hormone, has an additional regulatory role in amino acid metabolism [25]. Glucagon deficient mice exhibit modest perturbations of glucose control but marked hyperaminoacidemia [26]. Likewise, hyperglucagonemia due to glucagon-secreting tumours has modest effects on glucose but results in extreme hypoaminoacidemia [27,28]. These and later studies established that necrolytic migratory erythema due to hyperglucagonemia can be resolved by administration of amino acids [[28], [29], [30]]. Glucagon stimulates uptake of amino acids in studies on rat liver and hepatocytes [[31], [32], [33], [34]] and in human studies [[35], [36], [37], [38]], possibly through stimulation of branched chain 2-oxo acid dehydrogenase (BCKDH) [39] or stimulation of transaminases.

Finally, regulation of amino acids could play a role in actions of metformin on cardiac health, which remain poorly understood. It has long been known that metformin inhibits mTOR signaling, which is a pathway activated by amino acids including the BCAA leucine [40]. This pathway may be particularly relevant to metformin-dependent resolution of adverse anabolic remodelling of the heart, as administration of BCAA exacerbates hypertrophy in mice [41,42] and pharmacological inhibition of mTOR signalling by rapamycin inhibits this process [42,43]. In animal studies, metformin effects included suppression of (i) infarct size, (ii) adverse remodeling in diabetic and nondiabetic rodents [[44], [45], [46], [47], [48]] and (iii) heart failure progression in nondiabetic dogs [49]. Supportive genetic data for a critical role for BCAA in hypertrophy comes from studies on the transcription factor Kruppel-like factor 15 (KLF15), which acts as a ‘master regulator’ of amino acid catabolism in cells and in animals [50]. In mice, loss of KLF15 results in reduced BCAA catabolism and concomitant cardiac hypertrophy, including increased heart weight and exaggerated expression of hypertrophic genes [51]. KLF15 overexpression has also been found to ablate metformin suppression of glucose production in mice [52]. To date, there are few data translating these findings to humans but we have recently identified that the AA genotype in the KLF15 SNP rs9838915, is associated with increased risk of left ventricular hypertrophy (LVH), consistent with the loss of function phenotype in mice [53]. Despite this progress, the mechanism(s) underlying metformin's benefit in incidence of cardiovascular disease (CVD) remain unclear.

These considerations have led us in the current study to use multiple approaches, including human studies, to define the role of BCAA in therapeutic action of metformin.

## 2 Materials and methods

### 2.1 Cell-based studies

#### 2.1.1 Materials

Antibodies were used as previously described [54]. Briefly, the antibody to Ser79-phosphorylated acetyl-CoA carboxylase (ACC) was generously gifted by the MRCPPU at the University of Dundee. Total ACC, total AMP-activated protein kinase (AMPK)α, Thr172-phosphorylated AMPKα, total S6 ribosomal protein, Ser240/244-phosphorylated S6, Thr389-phosphorylated p70S6K and total p70S6K antibodies were from CST. Actin antibody was from Merck, leucine from Sigma, metformin from Calbiochem and phenformin from Sigma.

#### 2.1.2 Cell extraction, culture and lysis

Primary hepatocytes were extracted as described previously [54], by collagenase digestion. Briefly, pelleted hepatocytes were resuspended in M199-Glutamax media (ThermoFisher) supplemented with 100 μg/ml penicillin, 100 μg/ml streptomycin, 0.1% (v/v) BSA (ThermoFisher), 10% FBS (ThermoFisher), 10 nM Insulin Actrapid (Novo Nordisk 041–7642), 200 nM Triiodothyronine (Sigma T2877) and 500 nm Dexamethasone (Merck 265,005). Cell viability was measured using 0.04% Trypan blue stain and the cell number determined using a haemocytometer. Cell viability >90% was required to allow experimental use. After isolation, cells were maintained at 37 °C and 5% CO_2_ for 4 h, media replaced and used the following day. Hepatocytes were washed twice in warm PBS before treatment with the drug panel in EBSS (Sigma) or drug free EBSS (Basal) for 120 min, followed by refeeding for 60 min with MEM amino acids (Gibco) supplemented with 4 mM glutamine (Gibco).

Wild-type and mouse embryonic fibroblasts (MEFs) where the AMPK catalytic subunits are knocked out, were maintained in MEM (minimum essential media)-α supplemented with 10% FBS in T175 flasks. Experimental cells were seeded at a density of 450,000 cells per well in a 6-well plate and used as described above.

Cells were lysed by scraping into ice-cold lysis buffer: (50 mM Tris acetate pH7.5, 1% (w/v) Triton X100, 1 mM EDTA, 1 mM EGTA, 0.27 M sucrose, 50 mM NaF, 1 mM sodium orthovanadate, 10 mM β glycerophosphate, 5 mM sodium pyrophosphate, 1 mM benzamidine, 0.2 mM phenylmethylsulfonyl fluoride (PMSF) and 0.1% (v/v) β-mercaptoethanol) and then prepared for SDS-PAGE and immunoblotting as described previously [[55], [56], [57]]. Protein concentration was measured with Bradford reagent (for blots) and BCA (for uptake experiments).

Cardiac ventricular myocytes were prepared from neonatal mice (1–5 days) based on a protocol described earlier [58]. Briefly, ventricles from excised hearts were finely sliced and cardiac ventricular myocytes obtained by collagenase type II (Gibco) digestion (22.5 mg collagenase, in 50 ml of ice-cold HBSS no calcium, no magnesium). There were four digestions, one of 5 min and three for 10 min in a shaking water-bath at 37 °C. Myocytes and non-myocytes were separated by pre-plating for 2 h in 67% high glucose DMEM (Gibco 41966029), 17% M199+glutamax (Gibco 41150087), 10% horse serum, 5% fetal bovine serum and 2% penicillin/streptomycin. After this time the cells were plated on gelatin-coated cell culture dishes. The next day, the medium was changed to 75% high glucose DMEM, 17% M199, 5% horse serum, 0.5% fetal bovine serum, 2% penicillin/streptomycin. The cells were maintained in this media until carrying out the experiments. The EBSS protocol was as carried out on hepatocytes.

Primary mouse hepatocyte RNA was extracted using the Rneasy MINI KIT (Qiagen) according to the manufacturer's instructions. cDNA was synthesized using RQ1 Rnase-Free Dnase kit and ImProm-II Reverse Transcription System (Promega). Real-time (RT) PCR was carried out using the 7900HT Fast Real-Time PCR System and reagents (Applied Biosystems), using the following primer sets: LAT1 (SLC7A5) Mm00441516_m1, LAT2 (SLC7A8) Mm01318974_m1, LAT3 (SLC43A1) Mm01336378_m1, CAT1 (SLC7A1) Mm01219063_m1, CAT2 (SLC7A2) Mm00432032_m1, SNAT2 (SLC38A2) Mm00628416_m1 and TATA-binding protein (TBP) Mm01277042_m1. Cycling conditions were: 50 °C for 2 min, 95 °C for 10 min, followed by 40 cycles of 95 °C for 15 s and 60 °C for 1 min. Expression is expressed relative to TBP mRNA for hepatocytes (Applied Biosystems) using the 2-ΔΔCt method. Control samples were normalised to 1 and results for all experimental samples were graphed as relative expression compared to control.

#### 2.1.3 Saturable leucine, MeAIB and metformin transport

Saturable leucine uptake was measured in hepatocytes and Caco-2 cells, using a tracer-quench assay, with two solutions, following an earlier method [59]. One solution contained a low, non-saturable dose of substrate (Tracer) and another contained a high, saturable dose of substrate (Quench). Both contained a fixed concentration of tritiated leucine (Perkin Elmer). The uptake of the saturated quench solution treated tissue was subtracted from that of the tracer treatment uptake to calculate the saturable uptake of leucine. Hepatocytes were incubated with the drug panel in EBSS or drug free EBSS (Basal) for 150 min. Following 150 min incubation, radiolabelled leucine, diluted in tracer (10 μM cold leucine) or quench (10 mM cold leucine) in EBSS to a concentration of 0.17kBq/ml, was added to the cells for 10 min. Counts were performed on lysates and media. Counts in lysates directly reflect AA absorbed during the uptake phase. Media was harvested (500 μl) for counting, and cells lysed in NaOH (50 mM), 1% SDS lysis buffer and mechanically dissociated. Tracer content was measured by scintillation counting, normalised to the protein concentration. Saturable MeAIB and metformin uptake in hepatocytes were also measured similarly, with ^14^C radiolabelled (0.17kBq/ml) MeAIB and ^14^C radiolabelled (0.17kBq/ml) metformin (American Radiochemicals) substituting for leucine.

Caco-2 cells were maintained according to Natoli et al., [97] in DMEM supplemented with 1 mM sodium pyruvate, 100 μg/ml penicillin, 100 μg/ml streptomycin, 1X non-essential MEM amino acids supplemented with 4 mM glutamine, 10% (v/v) heat inactivated FBS, 250 ng/ml amphotericin B (Gibco) and maintained at low seeding density. Cells were subjected to mycoplasma testing and were negative.

To model intestinal absorption of amino acids, Caco-2 cells were seeded on permeable Transwell membrane supports (Corning) to allow cell polarization. Monolayer integrity was routinely investigated using visual analysis under a light microscope and deemed sufficient if TEER values remained above 500 mV. For experiments, media was replaced on both sides of the membrane. Drug and amino acid free EBSS was added to the basolateral pool and EBSS with/without drug added to the apical pool for 2 h. Next, the apical media was carefully removed and replaced with tracer or quench leucine +/− drug in EBSS plus cold leucine. Basolateral efflux (appearance in the media) was measured by scintillation counting.

#### 2.1.4 Puromycin incorporation assay

Anabolic amino acid incorporation into protein was measured by a puromycin incorporation assay as described previously [60]. The assay detects puromycin (an amino acid analogue) incorporation in newly synthesised protein. Cardiac ventricular myocytes were grown in serum free medium overnight and then amino acid withdrawal was performed in EBSS as for hepatocytes. Cells were treated with/without metformin for 2 h before and then refed with amino acids for 1 h and 1 μM puromycin was added for 10 min prior to lysis. In some experiments, Angiotensin II was used as a hypertrophic stimulus overnight. Cells were then washed with PBS, lysed and subjected to western blotting with mouse antipuromycin primary antibody (Kerfsat, EQ0001).

#### 2.1.5 Anchored colony growth assay

Using TSC2^+/−^ang1 sarcoma cells, which spontaneously form tumour-like colonies in culture, colony formation experiments were performed in 6-well plates (Nunc). Sterile 1% and 0.6% solutions of Noble agar (Becton Dickinson) and a stock solution of 2X DMEM plus 20% FBS were prepared. Soft agar was prepared through mixing of the DMEM solution and the 1% agar solution 1:1 v/v which was layered on top of a bottom layer of agar and used as a growth medium for the cells. Trypsinised cells were resuspended in the DMEM solution at a density of 80,000 cells per 1.5 ml, then diluted 1:1 with 0.6% Noble agar solution (42 °C) and added on top of the set base layer at 1.5 ml per well. Growth media was added with or without drug and replaced every three days to prevent drying of the plate. Colonies were stained with 3-(4,5-dimethylthiazol-2-yl)-2,5-diphenyltetrazolium bromide (MTT, Gibco) to allow automated colony counting on a Biorad Chemidoc XRS camera system using OpenCFU© 3.9.0 colony counting software.

#### 2.1.6 Glucose assay

Primary hepatocytes were isolated from WT C57BL/6 J mice. Glucose assay was made in glucose free DMEM with lactate (100 mM), pyruvate (1 mM) and pen/strep (1%). The stimulation of glucose production was with glucagon.

Primary mouse hepatocytes were treated with metformin (0.25 mM-2.5 mM) or vehicle. After 24 h of incubation period, 500 μl of media was collected and glucose concentration was determined by GAGO assay modified to 96 well plate. For amino acid supplementation, glucose production medium contained DMEM glucose free (D5030; Merck), 3.75 sodium bicarbonate, supplemented with 2 mM Glutamine, 10 mM lactate and 1 mM sodium pyruvate with or without Glucagon (100 ng/ml), Metformin (0.25 mM), leucine (0.8, 7, 15 mM), cells were incubated for 12 h at 37 °C and 5% CO2, 500 μl of medium was collected and glucose concentration was determined using glucose assay kit (GHK20, Merck) as described by the provider.

#### 2.1.7 DIA proteomics

DIA Proteomics was carried out essentially as described previously [61], following a published STAR protocol [62]. Prior to proteomic analysis, primary hepatocytes were cultured in DMEM (Gibco), supplemented with 100 μg/ml penicillin:100 μg/ml streptomycin (Gibco) and 100 nM of Dexamethasone (Merck 265,005). The cells were treated with 0.25 mM of Metformin (Sigma–Aldrich) or its vehicle (sterile PBS) for 24 h.

#### 2.1.8 Animal care

C57BL/6 mice were maintained under a 12 h:12 h light:dark cycle (holding room lights on at 06:00 and off at 18:00) at 22 ± 1 °C and 50% humidity. Mice had ad libitum access to standard chow diet (7.5% fat, 75% carbohydrate, and 17.5% protein by energy [RM1 diet; Special Diet Services]) and water. All animal care protocols and procedures were performed in accordance with current regulations and ethical approvals.

### 2.2 Clinical validation

#### 2.2.1 Population cohort study: metformin interaction with LVH risk SNP rs9838915

Patients were derived from the Genetics of Diabetes Audit and Research Tayside Scotland (GoDARTS) study, the methods and cohort details have been described in full previously [63]. In brief, the GoDARTS study includes 10,149 patients with type 2 diabetes (DM) and 8,157 controls without DM. Clinical characteristics are collected at baseline and electronic health records are linked using a unique patient identifier for blood samples, prescribing and clinical outcomes. We also linked these patients through the same identifier to the Tayside echocardiography database which contains over 100,000 clinically requested scans. Patients were fully genotyped using the Affymetrix platform as previously described.

In this study we included all DM cases and excluded non-DM controls. Patients were stratified by genotype related to Kruppel-like factor 15 (KLF-15) (rs9838915) which we have previously shown is significantly associated with echocardiographic left ventricular hypertrophy (LVH) [53]. Genotyping and quality control have been described previously [64]. We defined left ventricular hypertrophy (LVH) according to the American Society of Echocardiography (ASE) criteria [65] as previously described [64]. Patients were classified as non-LVH controls if they either had echocardiography performed that confirmed no LVH (LV wall septal and posterior wall thickness <1.2 cm) or had no clinically requested echo and had never had a heart failure hospitalisation.

Logistic regression was used to determine the association of KLF-15 genotypes (GG vs. GA vs. AA) with presence of LVH with adjustment for age, gender, diabetes status, systolic blood pressure and BMI. Interaction testing was performed to determine whether the association of rs9838915 with LVH was different in those patients using metformin at the point of recruitment into the GoDARTS study. We used Angiotensin Converting Enzyme (ACE) inhibitors or Angiotensin II Receptor Blockers (ARB) use as a control group. A 2-sided p value of <0.05 was considered significant and analysis was performed using R version 3.4.3.

#### 2.2.2 Randomized placebo-controlled study: metformin exposure and amino acid levels in nondiabetic heart failure patients

To clinically validate that metformin's effects on amino acids may be detected in humans, we investigated available plasma from patients who had participated in a double-blind, placebo-controlled study (www.clinicaltrials.gov: NCT00473876) that had evaluated the impact of metformin on IR and exercise capacity in non-diabetic patients with chronic heart failure (CHF) [66]. Every patient had provided written informed consent prior to participation in this study, which was approved by the East of Scotland Research Ethics Service. This current study measured amino acids in all of the extant plasma samples from the trial (38/62). No data were excluded. The trial consisted of non-diabetic insulin resistant chronic heart failure patients (mean age, 65.2 ± 8.0 years; male, 90%; left ventricular ejection fraction, 32.6 ± 8.3%; New York Heart Association class I/II/III/IV, 11/45/6/0) who were randomized to receive either 4 months of metformin (n = 23 this study, 2 g/day) or matching placebo (n = 15). Insulin resistance was defined by a fasting insulin resistance index (FIRI) ≥2.7. The effect of metformin on BCAAs and plasma glutamine level was examined by investigating changes from baseline to final visit after 4 months of drug treatment in the study

#### 2.2.3 Amino acid analysis

Reverse Phase-High Pressure Liquid Chromatography (RP-HPLC) (Hewlett Packard series 1050) was carried out on the human samples [66], using a Waters® Nova-Pak C18 Column, (Waters (WAT086344) 60 Å, 4 μm, 3.9 mm × 150 mm, 1/pkg) at 55 °C, permitting faster separation of Phenylisothiocyanate (PITC)-bound amino acids. Chromatography utilised Buffer A (150 mM Sodium Acetate, 0.5% Triethylamine (TEA), pH corrected to 6.4) and Buffer B, which is a 1:1 mix of Buffer A and acetonitrile.

Amino acids are separated due to varying binding affinities between the PTC-bound amino acids and the C18-bound silicon beads within the column. The separated amino acids were eluted from the column and the eluted fraction measured by UV spectrometry at 254 nm. Measurement of the area under these peaks, using ClarityLite® software, and comparison with a standard solution containing known concentrations of amino acids, was used to calculate the amino acid concentrations in human blood. Plasma Alanine was analysed as fold change from visit 1 for Placebo and Metformin using the retention area under curve values, due to lack of alanine standard. Blood plasma was centrifuged at 4 °C for 20 min at 13,000rpm and 50 μl of the resulting supernatant was transferred to a fresh tube. Prior to loading, samples underwent a derivatisation procedure to enable binding of PITC to amino acids. Briefly, Trifluroacetic acid (TFA) was used to remove the protective *tert*-butyloxycarbonyl group on amines, and as an ion pairing reagent, improving retention and separation of hydrophobic compounds within the column stationary phase. Dried samples were then washed and re-dried by re-suspension in a solution of Sodium acetate, methanol and Triethylamine (TEA) (2:2:1). Next, a solution of methanol, diH_2_O, TEA and PTC (7:1:1:1) was prepared and allowed to react for 15 min before drying at 45 °C in a speedyvac. A final 20 μl methanol wash was performed and then dried. Prior to loading, dried samples were re-suspended in a 5% acetonitrile solution in buffer A. Samples were injected at a volume of 40 μl and pumped through the column at a flow rate of 1.2 ml/min.

#### 2.2.4 Statistical analyses

Comparisons between groups were made by ANOVA post-hoc or paired t-testing using GraphPad Prism. Results in bar graphs are expressed as mean ± SEM. Differences were considered statistically significant if *P* was less than 0.05. ∗∗∗ denotes p < 0.001; ∗∗ denotes p < 0.01 and ∗ denotes p < 0.05. For studies on the plasma, statistical analyses of data were performed using GraphPad Prism and Spearman correlation coefficients were calculated using SPSS 14.1.

## 3 Results

### 3.1 Metformin suppresses mTOR signalling in hepatocytes and mouse embryonic fibroblasts (MEFs)

**Figure 1:**
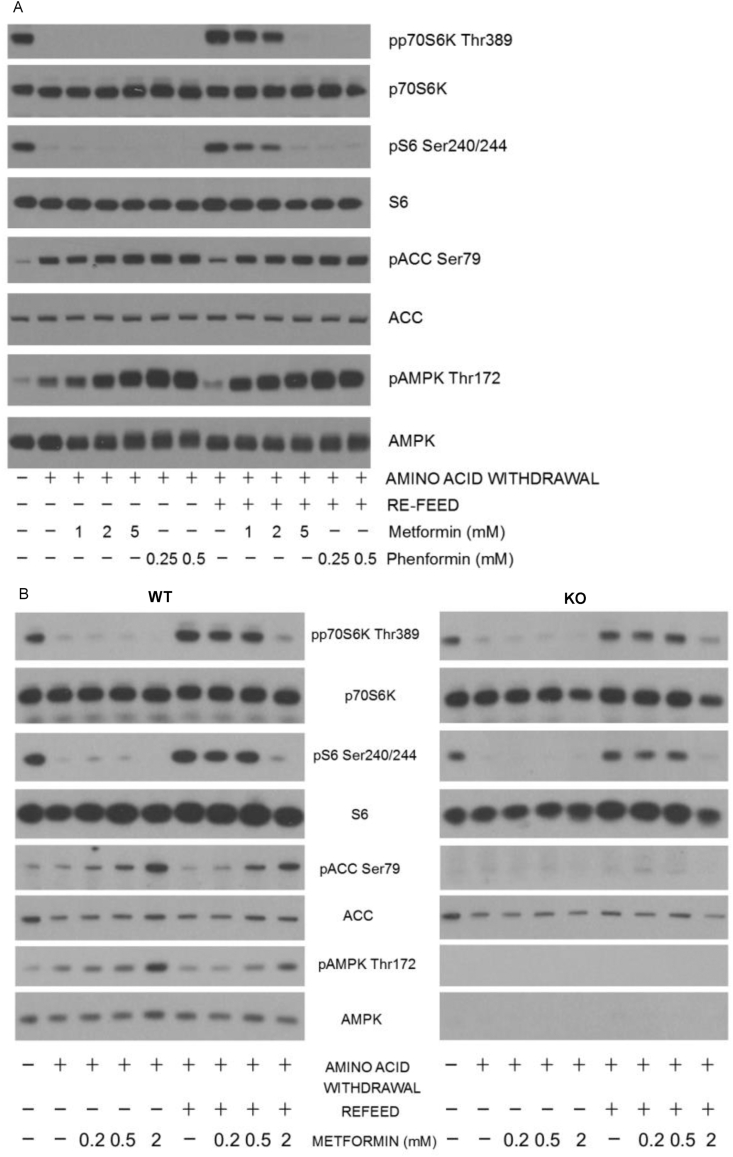
**Opposing effects of metformin and leucine on cell signaling.***(A)* Primary hepatocytes were starved of amino acids (2 h) followed by refeeding with/without 1X MEM amino acids supplemented with 4 mM l-glutamine for 60 min as shown, with or without the drugs shown present throughout the experiment. Cells were lysed and the lysates prepared for SDS-PAGE and immunoblotting. Effects on AMPK and mTOR signaling were measured using the antibodies shown, to study phosphorylation of p70S6K, S6, AMPK and ACC. *(B)* Wild-type (WT) and AMPK double knockout (AMPK KO) MEFs were treated as already described previously for hepatocytes, except that the MEFs had amino acids removed with/without drug for 15 h. N = 3 for each experiment.

Consistent with earlier data from serum-fed MEFs [67] and hepatocytes from fed animals [68], we found that millimolar concentrations of metformin and its more potent analogue phenformin suppressed mTOR signaling in response to amino acid refeeding in primary hepatocytes (Figure 1A, densitometry of blots is provided in Supplementary Figures. 1–5). We also carried out amino acid refeeding studies in MEFs and obtained similar results with metformin (Figure. 1B) indicating that the effect of metformin is not restricted to hepatocytes. In MEFs, metformin still suppressed the refeeding response in cells where both AMPK catalytic subunits are knocked out (Figure. 1B). We obtained similar data in hepatocytes lacking AMPK (Supplementary Figure. 6). Consistent with earlier high-dose metformin data from serum fed MEFs and hepatocytes from fed animals [67,68], these results indicate that the metformin-dependent suppression of amino acid-induced mTOR signaling observed, does not depend on AMPK signaling.

### 3.2 Investigation of the reversibility of inhibition of mTORC1 pathway by metformin and rapamycin

**Figure 2:**
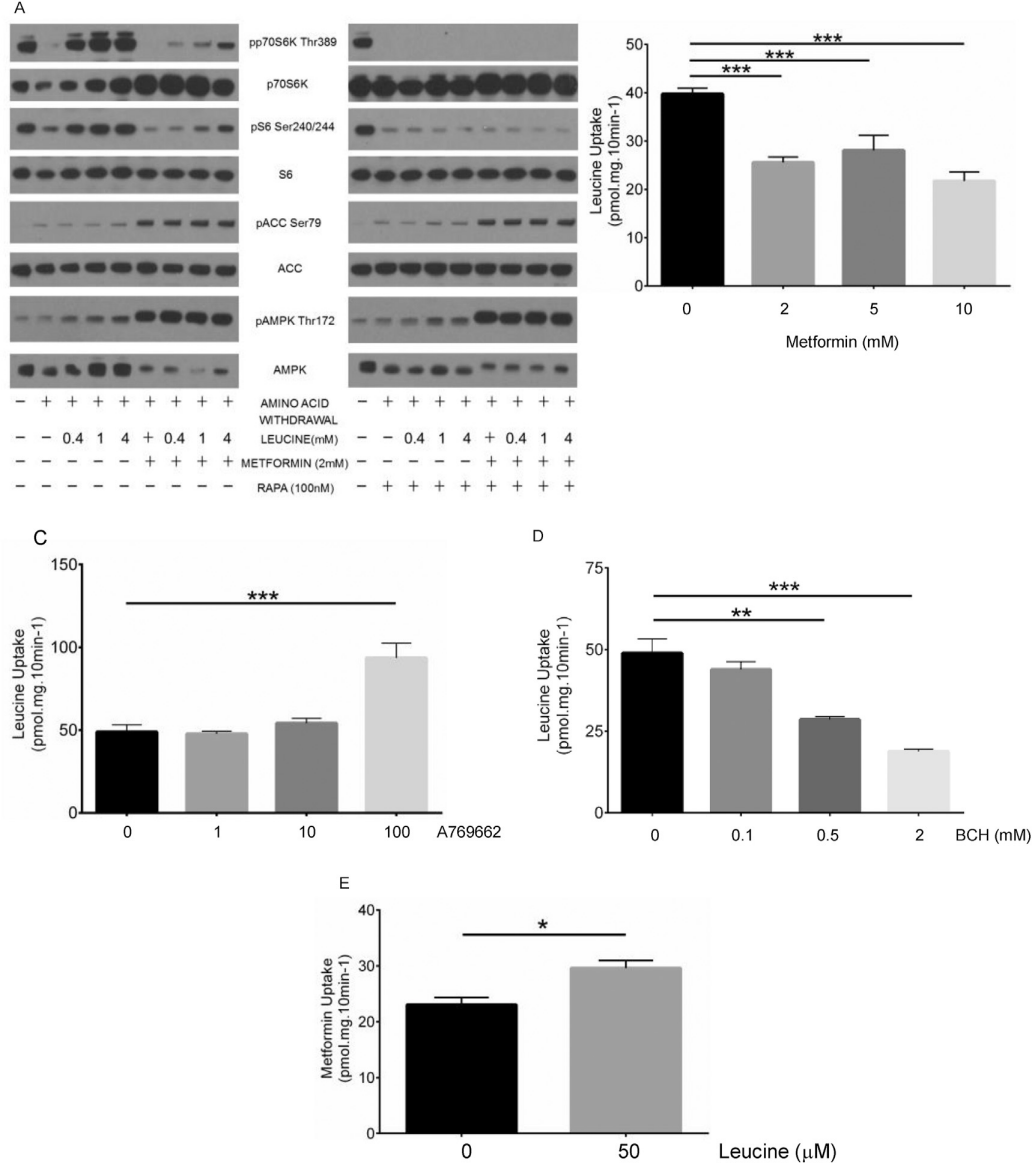
**Metformin suppresses leucine uptake and leucine selectively reverses effects of metformin but not rapamycin on mTOR signaling.***(A)* Primary hepatocytes were treated as in Figure 1A., except that they were supplemented with increasing concentrations of leucine 400 μM, 1 mM or 4 mM, with/without 2 mM metformin or 100 nM rapamycin (‘RAPA’) throughout the whole experiment. *(B-E)* Primary hepatocytes were pretreated for 150 min with metformin (B), A-769662 (C), 2-Aminobicyclo [2,2,1]heptane-2-carboxylic acid (BCH, D) and saturable leucine uptake was measured as described in the Methods. (E) Saturable metformin uptake was determined as described in the Methods, in the presence or absence of 50 μM added leucine. ∗∗∗ denotes p < 0.001 and ∗∗ denotes p < 0.01 with respect to basal. Each experiment was performed at least 3 times. Error bars are SEM. ∗ denotes p < 0.05 significant change between the columns marked, ‘ns’ denotes ‘not significant.’ N = 3 for each experiment.

Further analysis of cellular signaling in hepatocytes revealed that metformin-induced activation of AMPK signaling occurred irrespective of amino acid feeding and rapamycin-dependent inhibition of mTORC1 was also unaltered by increasing leucine supplementation (Figure 2A). In contrast, the suppressive effect of metformin on mTOR signaling could be blunted by increasing leucine concentrations. The reversibility of the metformin effect suggests attenuated but intact leucine sensing machinery. To examine the physiological significance of these findings, we compared antineoplastic effects of the drugs utilising the TSC2+/− ang1 cell line, which has been used to study suppressive effects of metformin and rapamycin on colony formation in soft agar [67]. Basal media contained 0.8 mM leucine and additional leucine supplementation increased colony formation (Supplementary Figure. 7). Consistent with its location distal of amino acid sensing, rapamycin reduced colony formation and strongly suppressed the response to amino acid supplementation, irrespective of leucine concentration (Figure 2C). With metformin, leucine increased colony formation but the slope of this increase was reduced, providing further evidence that metformin suppresses but does not abolish sensitivity to leucine (Figure 2C).

### 3.3 Metformin suppresses leucine uptake

We hypothesised that metformin may suppress amino acid uptake, which we measured using radiolabelled leucine. Metformin but not the specific AMPK activator A-769662, suppressed amino acid uptake similarly to 2-Aminobicyclo [2,2,1]heptane-2-carboxylic acid (BCH) (Figure 2D–F). BCH is an amino acid analogue that specifically inhibits amino acid transporters of system L family, which transports branched chain amino acids (BCAA, leucine, isoleucine, valine).

**Figure 3:**
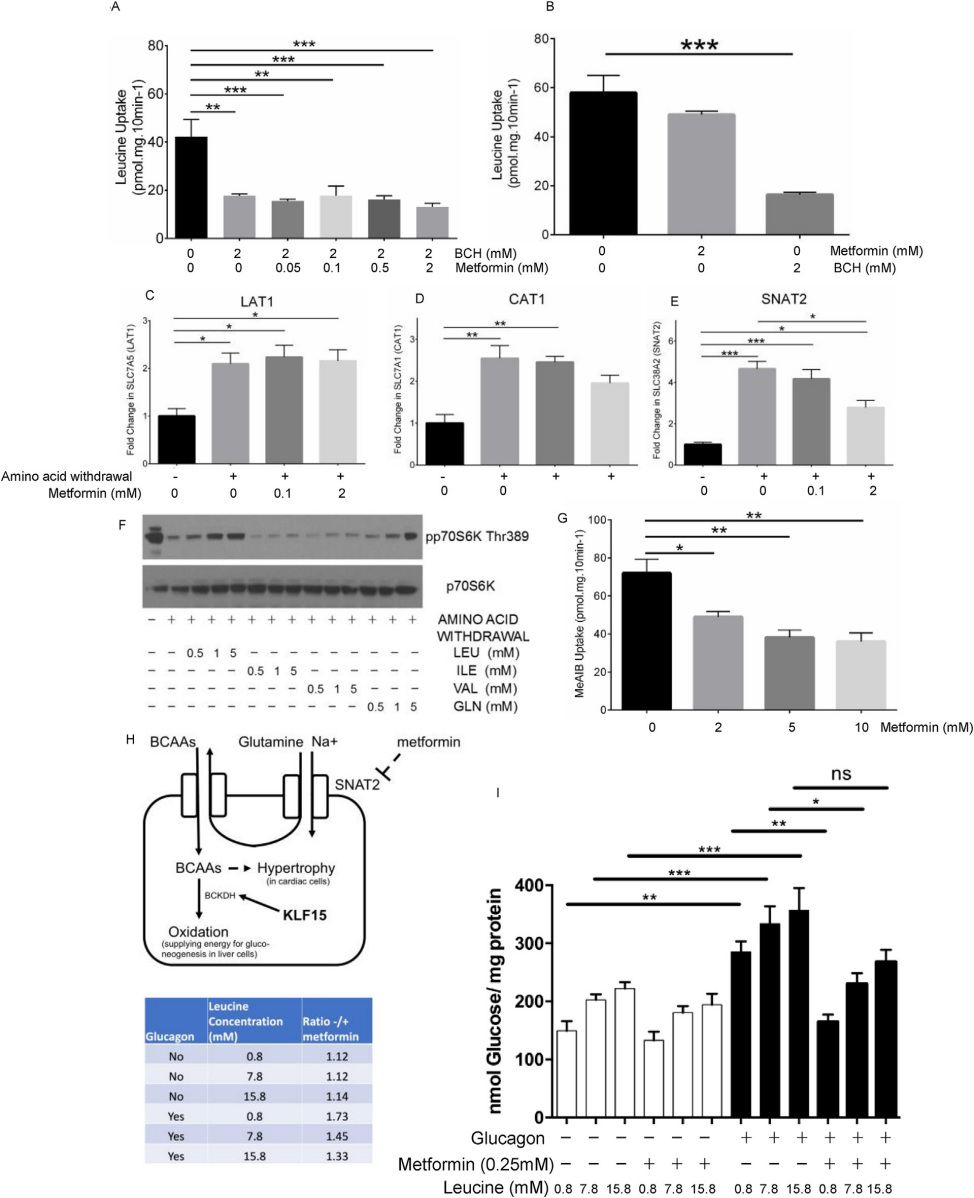
**Metformin suppresses function and expression of the glutamine transporter SNAT2.***(A)* Effect of adding metformin treatment in addition to BCH treatment (150 min for both agents). *(B)* Leucine uptake was determined as in Figure 3, except acute (10 min) effects of metformin and BCH were compared. *(C-E)* Cells were starved of amino acids for 3 h in the presence or absence of metformin as shown and gene expression of LAT1 (C), CAT1 (D) and SNAT2 (E) was measured by RTPCR as described in the Methods. *(F)* Primary hepatocytes were treated as in Figure 1, except that effects of single amino acids leucine, isoleucine, valine and glutamine on mTOR signaling were analysed. *(G)* Primary hepatocytes were pretreated for 150 min with metformin, and saturable MeAIB uptake was measured as described in the Methods. *(H)* Schematic of metformin and KLF15 actions on BCAA homeostasis. Metformin suppresses BCAA uptake by reducing functional SNAT2, which selectively inhibits mTOR activation by BCAA, in contrast rapamycin inhibits mTOR activation from all stimuli. These effects of metformin may limit the supply of amino acids for gluconeogenesis, or the supply of energy for gluconeogenesis in the liver. KLF15 also suppresses BCAA signaling, by inducing BCAA catabolism. *(I)* Effect of amino acid supplementation on glucose production with/without glucagon and metformin. The inset shows how fold change due to metformin is attenuated by adding amino acids Between columns, ∗∗∗ denotes p < 0.001, ∗∗ denotes p < 0.01, ∗ denotes p < 0.05 significant change. Each experiment was performed at least 3 times. Error bars are SEM.

In principle, metformin and leucine could compete for uptake at the same transporters, but physiological leucine concentrations stimulated metformin uptake (Figure 2G), moreover higher concentrations of leucine did not alter metformin uptake (data not shown). Co-treatment with metformin and BCH for 3 h did not have an additive suppressive effect on leucine uptake however, suggesting that both metformin and BCH suppress system L amino acid transport but that metformin acts indirectly (Figure 3A). Short term incubation (10 min) with metformin was much less effective than BCH at reducing leucine uptake however (Figure 3B, compare with Figure 2D,F). Together these data suggest that metformin does not directly compete with leucine on Large Neutral Amino Acids Transporter (LAT) 1–3 and likely alters leucine uptake by an intracellular mechanism.

### 3.4 Metformin selectively suppresses expression and activity of the SNAT2 glutamine transporter

We investigated the role of several transporter gene families in metformin's effects on amino acid refeeding. We found that amino acid withdrawal stimulated expression of leucine receptors LAT1-3 (Figure 3C and Supplementary Figures. 8A and B); however, metformin had little effect on expression of these genes, again consistent with an indirect mechanism of the drug on BCAA uptake.

CAT1 belongs to the y^+^ family that is responsible for uptake of arginine, which activates mTORC1 through interaction with the GATOR2 inhibitory complex CASTOR [69]. Like LAT1-3, CAT1 (SLC7A1) and CAT2 (SLC7A2) expression was enhanced by amino acid withdrawal. There was a modest trend towards reduced gene expression with metformin but this did not reach significance even at the highest concentration of the drug (Figure 3D and Supplementary Figure. 8C).

Glutamine acts on mTOR at least in part through feedback suppression of SNAT2, which will in turn suppress BCAA uptake because SNAT2 couples ‘uphill’ BCAA accumulation to the plasma membrane sodium gradient [70,71]. Like LAT1-3 and CAT1-2, SNAT2 expression was increased by amino acid withdrawal but in contrast to the other transporters, metformin strongly inhibited SNAT2 expression that had been induced by amino acid withdrawal, in an AMPK-independent manner (Figure 3E and Supplementary Figure. 8D). In support of this and consistent with our earlier data and that of others [70,71], we found that mTOR pathway signaling in hepatocytes was also stimulated by glutamine (Figure. 3F). To provide direct functional evidence that metformin acts on SNATs in hepatocytes we measured ^14^C radiolabelled MeAIB uptake in the presence and absence of chronic metformin treatment. MeAIB is a non-metabolisable substrate for system A transport. Consistent with our RTPCR data, we found that metformin suppressed MeAIB uptake (Figure 3G). A schematic for the proposed mechanism of amino acid regulation by metformin is in Figure 3H. We also investigated metformin's effect on glucose production, with and without amino acid supplementation. In this experiment, we found that additional amino acids increased glucose production (Figure 3I). In addition, higher concentrations of amino acids attenuated effect of metformin on glucagon (Figure 3I and inset).

### 3.5 Proteomic investigation of effect of metformin on liver cell amino acid transporters

**Figure 4:**
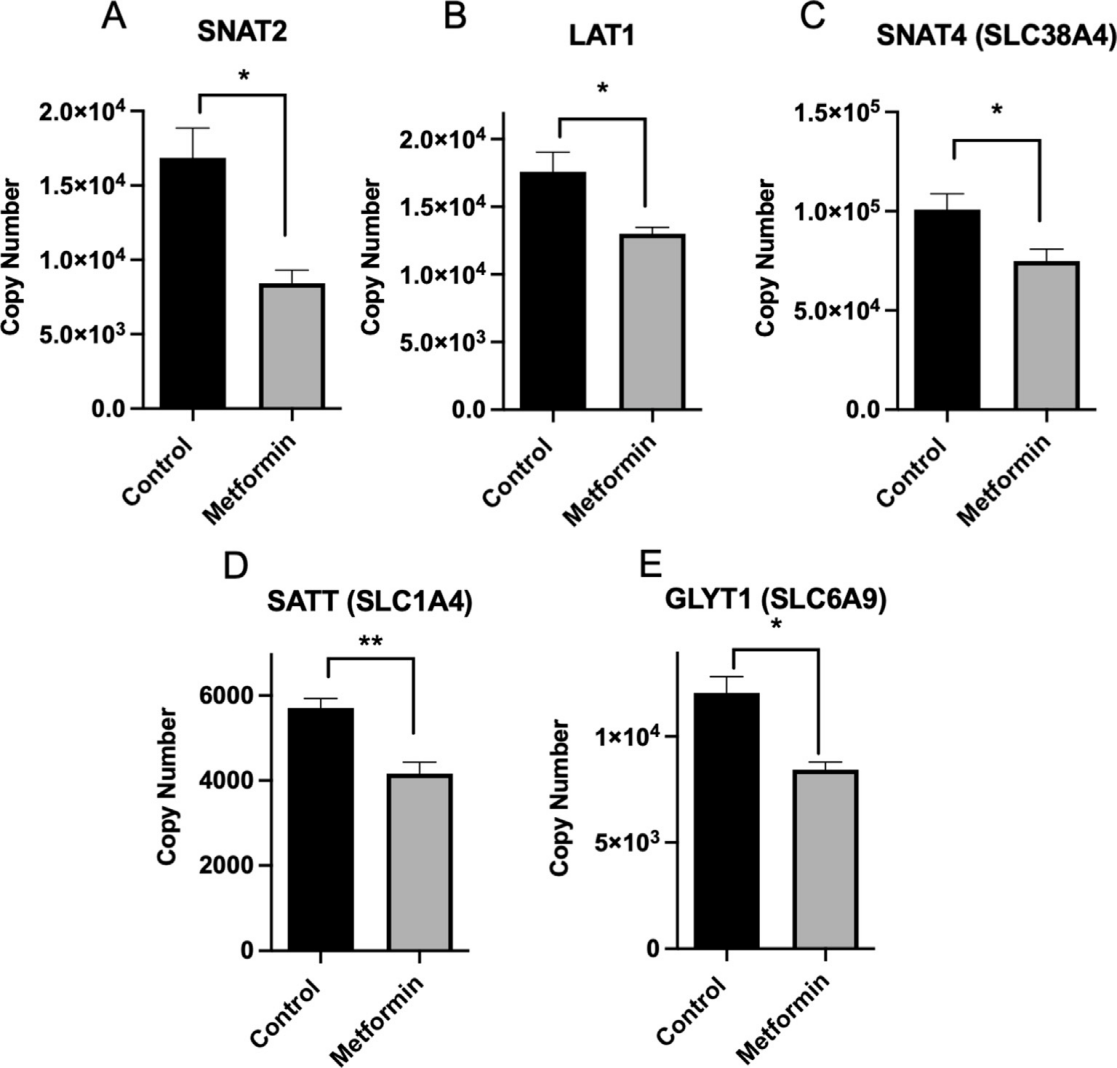
**Metformin selectively suppresses SNAT2, LAT1 and other amino acid trans-porters.***(A-E)* Liver cells were treated with/without 250 μM metformin for 24 h and DIA proteomics was carried out as described in the Methods. Amino acid transporters changed significantly by metformin are shown. Between columns, ∗∗ denotes p < 0.01, ∗ denotes p < 0.05 significant change. Each experiment was performed at least 3 times. Error bars are SEM.

We followed up our gene expression data by carrying out Data Independent Acquisition proteomics on liver cells, to determine which amino acid transporters were suppressed by 250 μM treatment for 24 h. Consistent with our gene expression data, SNAT2 was the most strongly suppressed amino acid transporter and we found that LAT1 and SNAT4 were also suppressed (Figure 4A–C). Metformin also suppressed SATT, which transports alanine, serine, cysteine and threonine [72] (Figure 4D), and GlyT1, a sodium and chloride dependent glycine transporter (Figure 4E).

### 3.6 Direct effects of metformin on mTOR signalling and amino acid incorporation into cardiac ventricular myocytes

**Figure 5:**
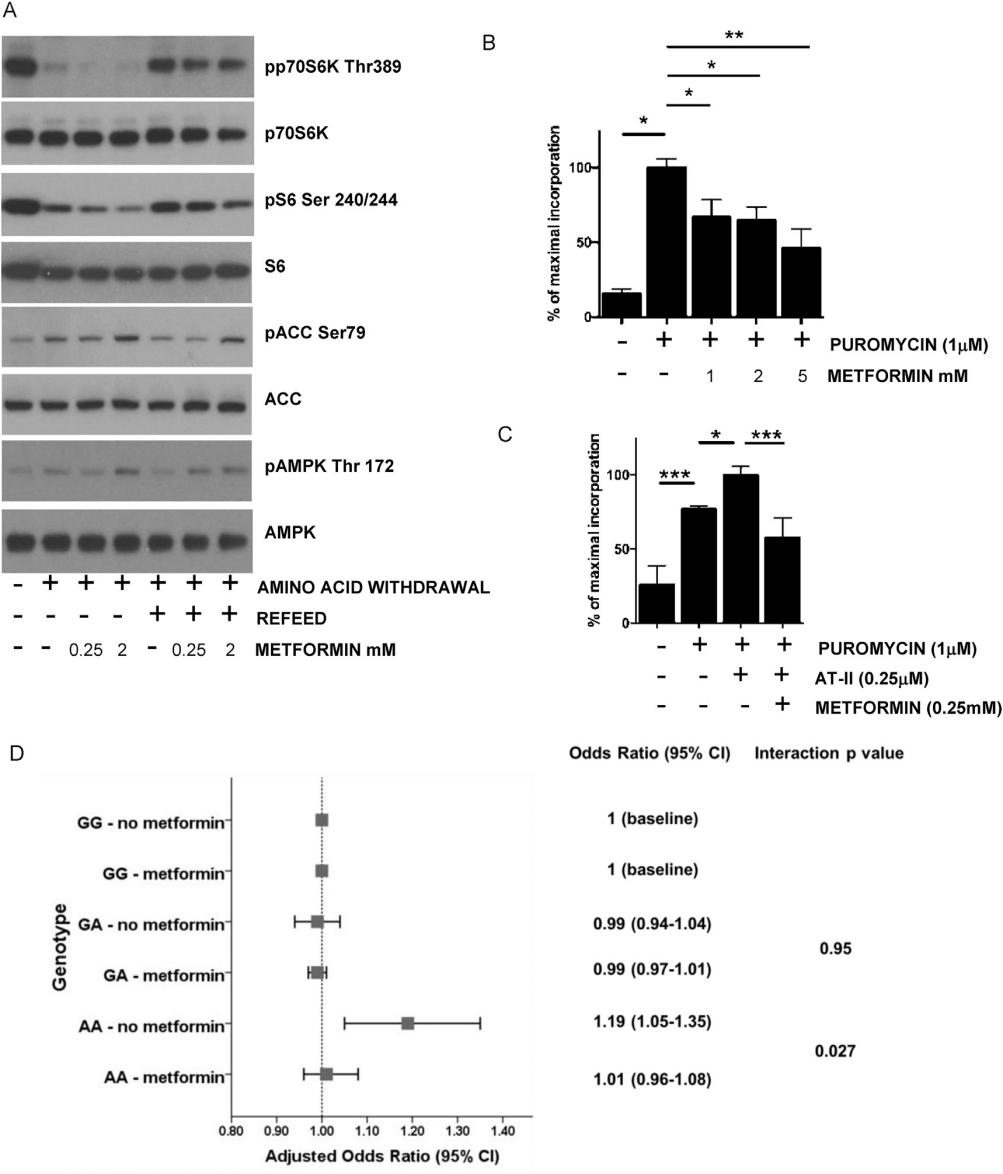
**Effects of metformin in cardiac myocytes.***(A)* Primary cardiac ventricular myocytes were starved of amino acids (2 h) followed by refeeding with/without 1X MEM amino acids supplemented with 4 mM l-glutamine for 60 min as shown, with or without the drugs shown present throughout the experiment. Cells were lysed and the lysates prepared for SDS-PAGE and immunoblotting. Effects on AMPK and mTOR signaling were measured using the antibodies shown, to study phosphorylation of p70S6K, S6, AMPK and ACC. *(B, C)* Primary cardiac ventricular myocytes were starved of amino acids for 2 h with or without metformin and refed with AA for 1 h prior to puromycin treatment for 10 min. Angiotensin II was used as a hypertrophic stimulus overnight (C). *(D)* Association of KLF15 genotype with LVH stratified by use of metformin. The significant association of the KLF15 AA genotype with LVH was completely attenuated in metformin users.

In the final part of our cellular study, we investigated whether metformin exerts direct effects on amino acid homeostasis in cardiac ventricular myocytes, as in an earlier randomised clinical trial, we had observed an anti-hypertrophic effect of metformin [73]. Consistent with the data in hepatocytes, metformin suppressed mTOR signaling in response to amino acid refeeding (Figure 5A). To study effects of metformin on anabolic incorporation of amino acids into protein in cardiac ventricular myocytes, we exploited a puromycin incorporation assay, previously used to study this in skeletal muscle [60]. Using this approach, we observed that metformin suppressed basal amino acid incorporation into cardiac ventricular myocytes (Figure 5B). In addition, metformin suppressed amino acids incorporation when a hypertrophic stimulus, angiotensin II, was applied (Figure 5C).

### 3.7 Effects of metformin on the KLF15 LVH risk allele in a large DM population cohort

We wished to identify a genetic test of the notion that modulation of BCAA by metformin is clinically important in therapeutic effects of the drug. SNAT2 knockout mouse pups die shortly after birth [74] and LAT1 knockout is embryonic lethal [75]. There is also much redundancy in the functionality of amino acid transporters. Consequently, a genetic test at the level of the transporters themselves is unlikely to be informative. In aggregate though, once taken up by the cell, amino acids may or may not be catabolised. If they are catabolised, then amino acids can provide energy or substrates for example for glucose production. If they are not catabolised, then they may be used for protein synthesis. This prompted us to investigate human SNPs, particularly of the transcription factor KLF15, which normally acts to promote BCAA catabolism, through activation of enzymes such as BCKDH and BCAT. In mice, KLF15 overexpression has previously been shown to attenuate metformin response [52]. In addition, in humans, we previously found KLF15 SNP rs9838915 increased LVH risk, consistent with loss of function, reduced BCAA catabolism and uninhibited hypertrophic amino acid signaling ([53] and see schematic Figure 3H). We reasoned that suppression of BCAA uptake by metformin would attenuate LVH risk in the AA allele of KLF15. To test for such an interaction, we used the GoDARTS diabetic cohort. We stratified patients by genotype and by exposure to metformin and in total, 7,146 patients were included. The mean age of the cohort was 64.8 ± 11.8 years and 3,902 (54.6%) were male while the majority of patients (5,774; 80.8%) were prescribed metformin. Baseline characteristics stratified by rs9838915 genotype are reported in Supplementary Table 1. In total, the minor allele frequency (A) was 18.4% (AA genotype 235 patients (3.3%), GA genotype 2,163 (30.2%), GG genotype 4,748 (66.4%)). There were no significant differences in age, gender, systolic blood pressure, duration of diabetes, metformin use, HbA1c or body mass index between the groups.

The presence of LVH was identified as previously described in 1,655 patients (23.2%) [64]. After adjustment for age, gender, systolic blood pressure there was a significant interaction between metformin use and the association of the rs9838915 AA genotype with LVH (p = 0.027) (Supplementary Table 2). Compared to GG genotype patients, there was no significant increase in likelihood of LVH in GA genotype patients. In contrast, patients with the AA genotype were significantly more likely to have LVH if they had never taken metformin (OR 1.19; 95% CI 1.05–1.35, p = 0.006); however, this association was not apparent in those who had used metformin (OR 1.01; 95% CI 0.96–1.08, p = 0.64) (Supplementary Table 2 and Figure. 5D). We included Angiotensin Converting Enzyme (ACE) inhibitor or Angiotensin II Receptor Blockers (ARB) use as a control group, to confirm a metformin-specific interaction. In these patients, there was no interaction with the genotype (Supplementary Table 2). Together, these results demonstrate that metformin attenuates the LVH phenotype of the AA risk allele of KLF15.

### 3.8 Effect of metformin on BCAA and glutamine plasma levels in humans with nondiabetic heart failure

**Figure 6:**
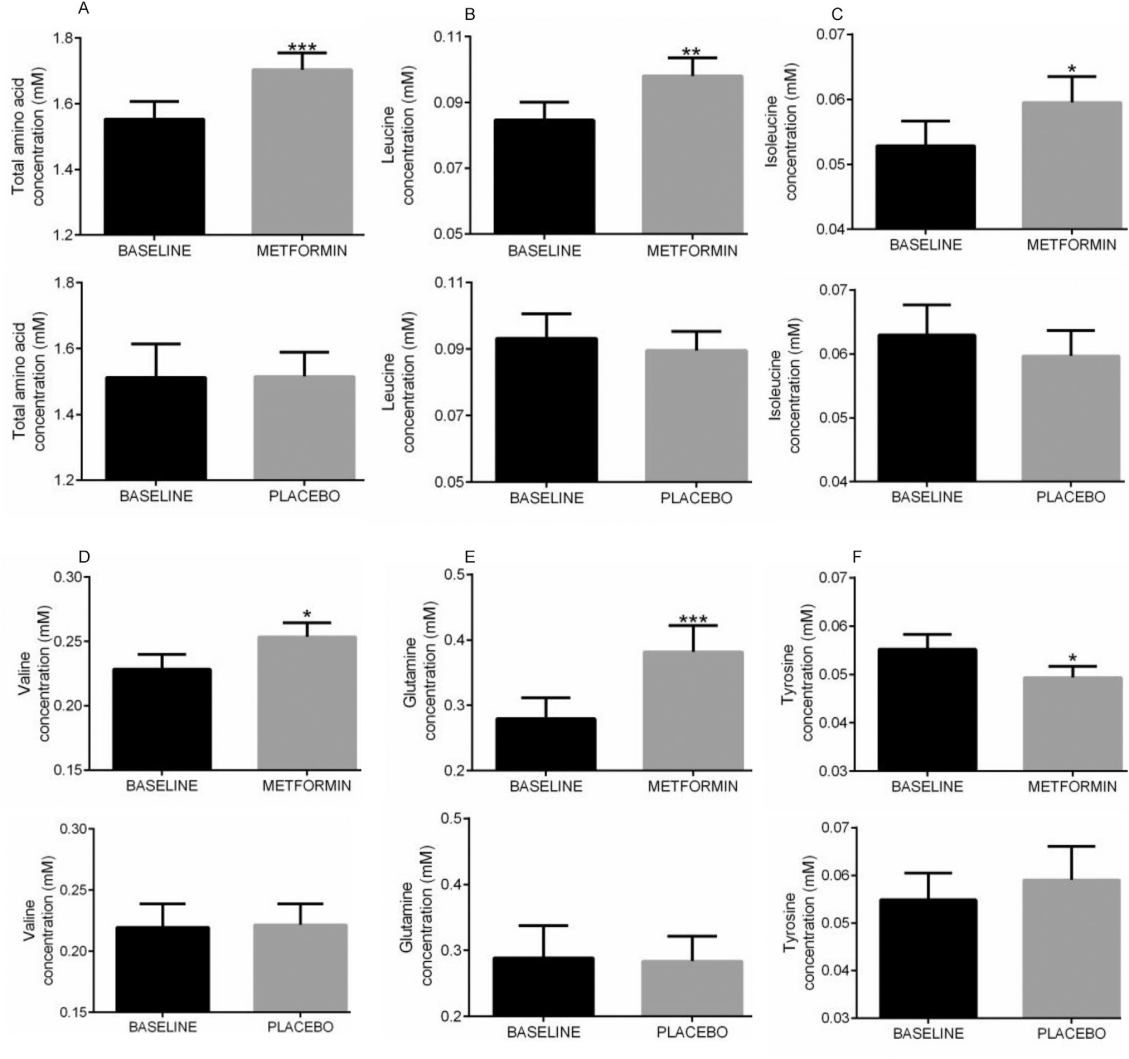
**Metformin causes plasma build-up of branched chain amino acids and glutamine in nondiabetic aged humans.***(A-F)* Plasma was obtained from non-diabetic aged humans before and after 4 months of treatment with metformin (n = 23, 2 g/day) or placebo (n = 15) as described previous-ly [37]. Plasma amino acid levels determined as described in the Methods are presented for total ami-no acids (A), leucine (B), isoleucine (C), valine (D), glutamine (E) and tyrosine (F). Other amino ac-ids are presented in supplementary material. ∗∗∗ denotes p < 0.001, ∗∗ denotes p < 0.01, ∗ denotes p < 0.05 between first and second sample.

Data on amino acids is not available for the GoDARTS cohort. To validate that metformin can selectively affect BCAA in humans, we were however able to utilise plasma samples collected during a RCT of metformin in non-diabetic heart failure patients [66]. Prior investigation of CACO intestinal cells had found no effect of metformin in saturable leucine basolateral efflux from intestinal cells (Basal 0.64 (95% CI 0.55–0.72) pmol/min, versus 10 mM metformin 0.63 (95% CI 0.58–0.67) pmol/min), suggesting that metformin is unlikely to affect amino acid uptake across the gut, despite the high concentrations of the drug residing in that tissue. We hypothesised that in such circumstances, suppression of amino acid uptake into tissues might lead to a build-up of circulating amino acids. Specifically, suppression of system A transporters such as SNAT2 would be expected to increase selectively, glutamine, leucine and other BCAA levels in the circulation. In the plasma samples from the clinical study, we did find altered amino acid profiles following 4 months of metformin treatment. All extant samples were analysed, no data being excluded. Plasma total amino acids increased (Figure 6A) but consistent with the evidence that SNAT2 is inhibited by metformin, this increase was almost entirely restricted to each BCAA, leucine (Figure 6B), isoleucine, valine (also transported by LAT1-3; Figure 6C,D) and in addition to these, glutamine, which was most robustly increased (Figure 6E). The gluconeogenic substrate alanine was also increased 13% (p = 0.019). Arginine was modestly but significantly increased (Supplementary Figure. 9), similar to the modest effects on CAT1 and CAT2 expression. No other amino acids measured were significantly increased (Supplementary Figure. 9). In addition to the amino acid increases already discussed, a modest decrease in tyrosine was observed (Figure. 6F). Baseline and changes in clinical parameters in these patients are presented in Supplementary Tables 3A and 3B In correlation analysis, increases in amino acids, particularly glutamine, were strongly correlated with metformin treatment, as were fasting glucose and insulin (Supplementary Table 4A). Further bivariate correlation analysis of those parameters altered by metformin found correlations between fasting insulin resistance index (FIRI) and leucine as well as glucose and leucine (Supplementary Table 4B).

## 4 Discussion

### 4.1 Metformin inhibits mTOR signaling by inhibiting BCAA uptake

Previous work attributed suppression of mTOR signaling by metformin to AMPK dependent [68,76,77] and independent [67,68,78] mechanisms, mostly through studies on mTOR pathway phosphorylation activated by serum or by animal feeding. These approaches do not separate effects of amino acid signaling from other stimuli, for example insulin, that are known to regulate the mTOR pathway [79]. In two cell types studied previously, MEFs [67] and primary hepatocytes [68], we studied the effect of metformin on regulation of mTOR signaling specifically when induced by amino acid treatment for 1 h after a 2 h amino acid starvation period. We acknowledge that amino acid levels would be unlikely to fall so low *in vivo* but we wished to study amino acid induction of the pathway in isolation, over time periods simulating human day-time feeding behaviour. Consistent with the earlier data [67,68], we found that millimolar concentrations of metformin suppressed mTOR signaling activation by amino acid refeeding, in both cell types. Previous studies provided evidence that AMPK independent effects of metformin on amino acids occur at these doses and we confirmed this by pharmacological means and through use of MEFs and hepatocytes lacking active AMPK.

### 4.2 Metformin suppresses leucine uptake in hepatocytes reversibly, by reducing the availability of functional SNAT2

In additional cell signalling studies we established that metformin but not rapamycin-dependent inhibition of mTOR could be attenuated by increasing leucine supplementation. The relevance of the reversibility of the metformin effect to cell outputs was first confirmed using a colony formation assay in soft agar [67]. Consistent with the cell signaling data, both drugs suppressed colony formation but only with metformin could leucine rescue colony formation, providing further evidence that metformin alone suppresses but does not abolish sensitivity to leucine.

We found no evidence for metformin acting directly on amino acid transporters but the effect of metformin was not additive with the inhibitor BCH, which does suppress leucine uptake directly. Together, these findings suggest that both drugs suppress system L amino acid transport but that metformin acts indirectly. Consistent with this, we found that system A transporter SNAT2 was the most strongly downregulated transporter gene we studied, with the leucine antiporter LAT1 and Na^+^-independent Cationic Amino Acid Transporter (CAT) 1 responsible for uptake of arginine each downregulated to a lesser degree. Studying the proteome, SNAT2 was the amino acid transporter most strongly suppressed by metformin and LAT1 was also suppressed, supporting the model of metformin modulating tertiary regulation of BCAA uptake. Glutamine is not the only amino acid transported by SNAT2 but it is the most abundant and recognised as a key participant in tertiary regulation [80] of branched chain amino acid (BCAA, leucine, isoleucine, valine) uptake, because SNAT2 couples ‘uphill’ BCAA accumulation to the plasma membrane sodium gradient [70, 71, 80]. In support of SNAT2 targeting driving the effects of metformin (see schematic Figure. 5A) and consistent with earlier data establishing tertiary regulation of leucine by SNAT2 [70,71,80], we found that mTOR pathway signaling in hepatocytes was also stimulated by glutamine. Consistent with our gene expression and proteomics data, we found that metformin suppressed SNAT activity, measured directly by a radiolabelled uptake substrate. Further work will be required to determine if metformin affects amino acid transporter activity indirectly through restricting energy supply. Together our findings strongly suggest that metformin selectively desensitizes mTOR to amino acids, in contrast to rapamycin, which inhibits mTOR signaling inputs more broadly (see schematic Figure 3H). This seemingly subtle difference might contribute towards the variety of adverse metabolic effects, such as insulin resistance, encountered with rapamycin but not metformin [81,82]. Equally, our identification of a mechanism, different from rapamycin, mediating effects of metformin on mTOR signalling, might foster further research into apparent longevity-extending properties of both drug classes in vertebrates [83,84].

### 4.3 Increasing leucine concentration attenuates effect of metformin on glucagon-induced glucose production

Although BCAA transamination in rodent liver and amino acid oxidation are usually low, previous work has shown that glucagon induces amino acid catabolism in high doses such as might be relevant to diabetes states in humans [38], [39], [98]. These studies showed that in addition to increased uptake of glucogenic amino acids, glucagon stimulates leucine oxidation [38]. Work on rat liver has shown that supplementation with amino acids augment glucose production [99]. Consistent with these previous studies, we found that increasing concentrations of leucine increased glucose production. In addition, we found that leucine attenuated the effect of metformin on glucagon-induced glucose production. This suggests that metformin may suppress glucagon-induced glucose production through its actions on amino acid uptake, possibly by suppressing energy supply derived from BCAA oxidation, or from muscle-derived branched chain ketoacids and/or by limiting provision of gluconeogenic substrates, such as alanine.

### 4.4 Effect of amino acid concentration on metformin dose response

The appropriate concentration of metformin to use in cell culture is debated. Higher concentrations of metformin, in the micromolar or millimolar range, are usually required in short-term treatment to observe effects of the drug in cell culture, although lower concentrations are effective in longer term treatment (∼24 h), probably because metformin uptake into cells is slow [11]. Therapeutic metformin plasma concentration is generally understood to be in the low micromolar range, although concentrations as high as 0.9 mM have been reported in random tests of plasma metformin concentration in patients [85]. Our finding that increasing amino acid concentration suppresses metformin response on glucagon-induced glucose production and on signaling, might partly account for the discrepancy between effective doses *in vivo* and *in vitro*, as supraphysiological amino acid concentrations are invariably present in media. We focused on BCAA and further work will be required to determine whether other amino acids ablate metformin response. We do not rule out either that other medium components besides amino acids might contribute, as well as treatment duration, as *in vivo* studies tend to involve repeated dosing, whereas cell studies usually depend on short term dosing. There are also species-specific differences in metformin transporter expression, for example human intestinal expression of OCT1 may be lower than in mice [86]. These complexities highlight the importance of cross-referencing preclinical studies with clinical data, as we have done, wherever this is possible.

### 4.5 BCAA metabolism contributes to beneficial effects of metformin on LVH

Beneficial effects of metformin on CVD have been known for decades without much mechanistic insight. In an earlier randomised clinical trial for example, we observed an anti-hypertrophic effect of metformin in LVH [73] and this prompted investigation of whether metformin affected amino acid homeostasis in cardiac ventricular myocytes. We found that metformin suppresses mTOR signalling and anabolic incorporation of amino acids into proteins in these myocytes. Importantly, metformin suppressed amino acid incorporation induced by the hypertrophic stimulus, angiotensin II, a potent stimulus of cardiac hypertrophy which acts in a rapamycin-dependent manner [87]. These findings offer a plausible explanation for our earlier clinical observation and at a modest dose of drug. Given the ongoing discussion about appropriate dosing of metformin in rodent studies we strongly believe that a robust method of cross-validation of rodent cell findings, wherever possible, is to identify clinical correlates, as highlighted in our earlier multi-disciplinary work on anti-inflammatory actions of metformin [54]. Our earlier work on KLF15 also afforded an opportunity to perform a genetic test of the role of amino acids in metformin therapy, as SNAT2 knockout is embryonic lethal and amino acid transporters exhibit considerable amounts of redundancy. In earlier studies in mice, loss of the transcription factor KLF15 resulted in cardiac hypertrophy, including increased heart weight and exaggerated expression of hypertrophic genes [51], at least in part through controlling the activity of other key transcription factors including GATA, MEF2 and myocardin [88]. These effects are mediated by reduced BCAA catabolism in these animals. Consistent with these findings, in a large-scale genotyped GoDARTS DM cohort, we replicated our earlier finding that the AA rs9838915 genotype of KLF15 increases risk of LVH in humans, suggesting loss of function and disinhibited hypertrophic amino acid signaling ([53] see schematic Figure 3H). We reasoned that suppression of BCAA uptake by metformin would attenuate this LVH risk allele and indeed when we stratified AA patients by exposure to metformin, those receiving metformin were at no more risk of LVH than any other genotype, whilst those not receiving metformin were at increased risk of LVH. We included Angiotensin Converting Enzyme (ACE) inhibitor or Angiotensin II Receptor Blockers (ARB) use as a control group, to confirm a metformin-specific interaction with the genotype. Together, these results strongly suggest that suppression of BCAA uptake by metformin attenuates LVH risk in humans.

### 4.6 Metformin increases BCAA and glutamine plasma levels in aged nondiabetic subjects with CVD

The GoDARTS cohort does not itself have data on plasma amino acids and in addition its observational nature means it is vulnerable to confounding, prompting us to further confirm selectivity of effects of metformin on BCAA in a RCT of metformin in a group of nondiabetic insulin-resistant heart failure patients. This allowed us to study effects of metformin on amino acids in aged humans, without selection bias or diabetes as potentially confounding factors. Investigation of intestinal cells suggested that metformin does not affect amino-acid uptake across the gut and there is in any case substantial absorption of dipeptides and polypeptides across the gut wall, irrespective of any possible effects on amino acid transporters. In such circumstances, suppression of aminoacid uptake might be expected to lead to a build-up of circulating amino acids and this is exactly what we found. Consistent with the evidence that SNAT2 is inhibited by metformin, this increase was mainly restricted to each BCAA and glutamine, which was the most robustly increased of all. We attribute a minor effect on arginine to the modest effects on CAT1 and CAT2 expression, supporting the idea that metformin might also act weakly on this arginine sensing mechanism in humans. Alanine, a gluconeogenic substrate, was also increased consistent with previous findings [89] and possibly indicative of gluconeogenic inhibition. In addition, glutamine is thought to make a significant contribution to glucose production in humans and this conversion is increased in type 2 diabetes, more so than alanine. Glutamine uptake is also stimulated by glucagon [33]. Consequently, inhibition of uptake of this amino acid by metformin might also contribute to inhibition of glucagon-induced glucose production [90], or indirectly, through suppression of anaplerotic mechanisms. Further work on these amino acids in relation to metformin action, will be required.

No other amino acids measured were significantly increased, strongly suggesting that effects of metformin on glutamine and BCAA transport that were the salient changes in cells are also critical for the effect of metformin on amino acids in this nondiabetic cohort. A previous study of metformin in coronary heart disease found that metformin tended to raise BCAA levels in plasma for up to 1 year but those changes were non-significant [89], perhaps because that work studied a much more heterogeneous patient group, including several kinds of heart disease and with observations at 18 months, when the effect of drug on BCAA levels seems to diminish [89].

Our proof of concept trial was not powered to investigate clinical outcome but we have shown earlier in these patients that metformin treatment significantly decreased fasting insulin resistance index (FIRI, from 5.8 ± 3.8 to 4.0 ± 2.5, P < 0.001) [66]. In correlation analyses, increases in amino acids, particularly glutamine, were strongly correlated with metformin treatment, as were fasting glucose and insulin. Further bivariate correlation analysis of these parameters found correlations between FIRI and leucine as well as glucose and leucine. Together, these results demonstrate that metformin's effects on amino acids strongly correlate with key aspects of the improving metabolic picture in these non-diabetic patients.

In addition to the amino acid increases already discussed, a modest decrease in tyrosine was observed which is unexplained by our model. In an earlier study, longer durations of treatment produced a larger effect of metformin in decreasing tyrosine [89]. It is possible that the observed decreases in tyrosine are due to increases in use of this amino acid as a fuel. Metformin has also been shown previously to increase plasma levels of alanine, which is a glucogenic amino acid [89], probably by directly sparing alanine from use as a glucogenic substrate. Our data concurred with that finding. Further work will also be required to determine how metformin mediates suppression of SNAT2, which may be related to observations that metformin acts on the v-ATPase–Ragulator complex [91] and might account for other observations that BCAA and glutamine oxidation are suppressed by metformin in tumour cells [92].

We acknowledge some limitations in our study. Limitations inherent in observational nonrandomized observational cohort data mean it was impossible to account for all possible confounding influences that may have biased our observed differences between groups. In addition, the functional effect of the rs9838915 risk allele of KLF15, which is intronic, has not been established experimentally [53]. Our observational results are consistent with knockout studies in rodent models [51] suggesting that the rs9838915 AA genotype confers reduced BCAA catabolism, which is then relieved by metformin suppressing BCAA uptake. The signal we identified of metformin increasing circulating amino acids in a randomized double-blind placebo-controlled trial, does however provide definitive evidence of amino acid regulation by metformin in the absence of DM. Whilst that data points towards a mechanism involving BCAA being important in humans, it does not confirm the mechanism. Our observations also suggest that more than one cell type may contribute to effects of metformin on amino acids and might also contribute towards previously observed association of DM with elevated plasma BCAA [93]. In terms of LVH though, we have made key observations in cardiac ventricular myocytes. Our use in these cells of the puromycin incorporation assay to directly measure reduced amino acid incorporation in the presence of metformin, provides a plausible mechanism for our earlier clinical observation of metformin reducing LVH in a human RCT [73]. Finally, because of the small size of the clinical trial, our proof-of-concept study was designed and powered only to investigate the study-specific end point of peak oxygen uptake in patients with heart failure and not clinical outcome. However, in addition to the observational data presented here, we have previously shown in a large population-based cohort study that patients with DM and heart failure treated with metformin alone or in combination with sulfonylureas were at significantly lower risk of all-cause mortality during 1 year and long-term follow-up than those who were treated with sulfonylurea alone [94]. Our findings on metformin and amino acids will now similarly need to be confirmed in other patient cohorts.

## 5 Conclusion

In conclusion, we have used cell studies to provide evidence that the well-tolerated type 2 diabetes drug metformin inhibits mTOR signaling by a different mechanism than rapamycin, through suppressing BCAA and glutamine uptake. The inhibitory effect of metformin is mediated, at least in part, by suppression of amino acid transporters. Amino acid supplementation attenuates many cellular responses to metformin. We demonstrated that the effect of metformin on BCAA uptake is clinically significant, as in a large cohort of genotyped DM patients, metformin treatment attenuates the LVH risk genotype of KLF15, a transcriptional ‘master regulator’ of BCAA catabolism. Furthermore, we demonstrated a selective effect of metformin on BCAA and glutamine in a double-blind placebo-controlled trial of non-diabetic heart failure patients. We conclude that metformin modulates amino acids in cell models and in humans. Our findings support an unfolding strategy of investigating specific mechanisms to determine their utility in treating specific genotypes for metformin prescription [96], as suggested recently for all diabetes drugs [95].

## Data Availability

Data will be made available on request.
