# Supplementary material for: Amino acid homeostasis is a target of metformin therapy
Source: Mol Metab. 2023 Jun 9;74:101750. doi: 10.1016/j.molmet.2023.101750 (PMC10328998; doi:10.1016/j.molmet.2023.101750)
Supplement: Multimedia component 1 [file mmc1.docx]

**Supplementary Material**

**Supplementary Table 1. Baseline Characteristics Stratified by rs9838915 genotype**

|  | GG (n=4,748) | GA (n=2,163) | AA (n=235) | p value between groups |
| --- | --- | --- | --- | --- |
| Age | 64.8 ± 11.9 | 64.9 ± 11.6 | 66.1 ± 11.8 | 0.28 |
| Male | 2,593 (54.6) | 1,183 (54.7) | 126 (53.6) | 0.96 |
| Systolic blood pressure (mmHg) | 143 ± 32 | 145 ± 33 | 143 ± 31 | 0.49 |
| Duration of diabetes (years) | 10.2 ± 7.0 | 10.2 ± 7.2 | 9.0 ± 5.5 | 0.47 |
| Metformin use | 3,824 (80.5) | 1,760 (81.4) | 190 (80.9) | 0.75 |
| ACE/ARB use | 2,717 (57.2) | 1,190 (55.0) | 142 (60.4) | 0.11 |
| Mean HbA1c | 58.7 ± 17.1 | 58.8 ± 17.9 | 57.0 ± 13.8 | 0.77 |
| Body Mass Index (kg/m^2^) | 29.7 ± 5.7 | 30.0 ± 5.7 | 28.8 ± 5.5 | 0.86 |

**Supplementary Table 2. Multivariable Logistic Regression for Association of rs9838915 genotype with LVH**

|  | Non-Metformin Users | | | Metformin users | | | Interaction p value |
| --- | --- | --- | --- | --- | --- | --- | --- |
|  | Estimate | SE | p value | Estimate | SE | p value |  |
| GG genotype | Baseline |  |  | Baseline |  |  |  |
| GA genotype | -0.012 | 0.025 | 0.63 | -0.110 | 0.012 | 0.36 | 0.95 |
| AA genotype | 0.176 | 0.064 | **0.006** | 0.015 | 0.031 | 0.64 | **0.027** |
|  | Non-ACE/ARB users | | | ACE/ARB users | | |  |
| GG genotype | Baseline |  |  | Baseline |  |  |  |
| GA genotype | -0.005 | 0.015 | 0.76 | -0.019 | 0.015 | 0.22 | 0.30 |
| AA genotype | 0.061 | 0.040 | 0.13 | 0.031 | 0.038 | 0.40 | 0.60 |

**Supplementary Table 3 A**

|  | Baseline | |  |
| --- | --- | --- | --- |
|  | Placebo (n=15) | Metformin (n=23) | *p-value* |
| Sex | 14M; 1F | 19M; 4F |  |
|  | Average (SD) | Average (SD) |  |
| Age (years) | \| 65.4 (7.08) \|  \|  \|  \| \| --- \| --- \| --- \| --- \| \|  \|  \| | \| 62.7 (7.62) \| \| --- \| \|  \| | 0.273 |
| **Metabolism Parameters**  BMI (kg/m^2^)  Insulin (mU/L)  Glucose (mmol/L)  FIRI | 29.18 (4.54)  20.71 (11.19)  5.21 (0.39)  4.33 (2.40) | 30.15 (4.86)  27.24 (14.05)  5.53 (0.65)  6.22 (3.75) | 0.547  0.132  0.065  0.073 |
| **Severity of Heart Failure**  BNP (pg/mL)  Ejection  Fraction (%) | 202.71 (280.00)  30.33 (8.68) | 125.47 (181.32)  34.93 (8.13) | 0.369  0.167 |
| **Haemodynamic Parameters**  Resting Systolic BP (mmHg)  Resting Diastolic  BP (mmHg)  Resting HR (bpm)  Peak VO2  VE/VCO2 slope  Total Exercise  Duration (s) | 117.07 (18.51)  74.60 (10.37)  73.53 (19.41)  17.64 (4.99)  32.10 (6.46)  898.27 (312.00) | 112.13 (13.44)  71.78 (9.80)  70.22 (14.60)  19.51 (4.14)  31.44 (6.16)  1062.39 (181.04) | 0.397  0.424  0.589  0.252  0.775  0.089 |

**Supplementary Table 3A: Metabolic, haemodynamic and other characteristics of subjects at baseline.**

**Supplementary Table 3 B**

|  | Change after 4 months treatment | |  |
| --- | --- | --- | --- |
|  | Placebo (n=15) | Metformin (n=23) | *p-value* |
|  | Average (SD) | Average (SD) |  |
| **Metabolism Parameters**  BMI (kg/m^2)^  Insulin (mU/L)  Glucose (mmol/L)  FIRI | 0.24 (0.90)  2.83 (6.10)  0.19 (0.69)  0.84 (1.44) | -0.79 (1.01)  -5.89 (7.41)  -0.35 (0.47)  -1.74 (2.12) | 0.003**  <0.001***  0.017*  <0.001*** |
| **Severity of Heart Failure**  BNP (pg/ml)  Ejection  Fraction (%) | -20.65 (106.13)  1 (10.61) (n=13) | -12.49 (64.43)  4.18 (13.42) (n=21) | 0.799  0.485 |
| **Haemodynamic Parameters**  Resting Systolic BP (mmHg)  Resting Diastolic  BP (mmHg)  Resting HR (bpm)  Peak VO2  VE/VCO2 slope  Total Exercise  Duration (s) | -6.33 (15.48)  -4.87 (9.35)  -0.53 (11.81)  1.78 (4.18)  6.25 (15.87)  8.53 (85.20) | -2.43 (12.76)  -1.78 (6.39)  3.96 (21.62)  0.13 (2.88)  -1.97 (8.15) (n=22)  -33.65 (98.86) | 0.438  0.041*  0.427  0.210  0.090  0.183 |

**Supplementary Table 3 B: Change in metabolic, haemodynamic and other characteristics of subjects at end of study.**

**SUPPLEMENTARY TABLE 4A**

| **Parameter** | **Correlating ∆Parameter/∆Amino Acid** | **r_s_ value** | **Number of patients** | ***P* value** |
| --- | --- | --- | --- | --- |
| ***Metformin Treatment*** | Insulin | -0.523 | 38 | 0.001*** |
|  | Glucose | -0.453 | 38 | 0.004** |
|  | FIRI | -0.562 | 38 | <0.001*** |
|  | Glutamine | 0.454 | 38 | 0.004** |
|  | Leucine | 0.405 | 38 | 0.012* |
|  | Total Amino Acid | 0.439 | 38 | 0.006** |

**A: Spearman’s bivariate correlation analysis between 4 months metformin treatment and accompanying changes in clinical parameters/plasma amino acids.** A negative value indicates a negative correlation between treatment and the clinical parameter (treatment lowers parameter). FIRI (Fasting Insulin Resistance Index). ∆parameter/ amino acid= change from Baseline to End of Treatment (4 months).

**SUPPLEMENTARY TABLE 4B**

| **∆Parameter/Amino Acid** | **Correlating ∆Parameter/∆Amino Acid** | **r_s_ value** | **Number of patients** | ***P* value** |
| --- | --- | --- | --- | --- |
| ***Insulin*** | **Leptin**  **Glucose**  **FIRI** | 0.428  0.573  0.953 | 38  38  38 | 0.007**  <0.001***  0.000** |
| ***Glucose*** | **Leptin**  **Insulin**  **FIRI**  **Lean mass**  **Leucine** | 0.353  0.573  0.757  0.345  -0.366 | 38  38  38  37  38 | 0.030*  <0.001***  <0.001***  0.036*  0.024* |
| ***FIRI*** | **Leptin**  **Insulin**  **Glucose**  **Lean mass**  **Leucine** | 0.451  0.953  0.757  0.389  -0.337 | 38  38  38  37  38 | 0.004**  <0.001***  <0.001***  0.017*  0.039* |
| ***Glutamine*** | **Arginine**  **Total Amino Acid** | 0.393  0.767 | 38  38 | 0.015*  <0.001*** |
| ***Leucine*** | **Glucose**  **FIRI**  **Arginine**  **Tyrosine**  **Valine**  **Isoleucine**  **Total Amino Acid** | -0.366  -0.337  0.464  0.465  0.817  0.855  0.592 | 38  38  38  38  38  38  38 | 0.024*  0.039*  0.003**  0.003**  <0.001***  <0.001***  <0.001*** |
| ***Total Amino Acid*** | **Glutamine**  **Arginine**  **Tyrosine**  **Valine**  **Isoleucine**  **Leucine** | 0.767  0.572  0.421  0.567  0.466  0.592 | 38  38  38  38  38  38 | <0.001***  <0.001***  0.008**  <0.001***  0.005**  <0.001*** |

#### **B: Spearman’s bivariate correlation analysis with parameters changed by 4 months metformin treatment.** A negative value indicates a negative correlation between treatment and the clinical parameter (treatment lowers parameter). FIRI (Fasting Insulin Resistance Index). ∆parameter/ amino acid= change from baseline to 4 months treatment.

**Supplementary Figure Legends**

**Supplementary Figure 1. Densitometry of blots in main figure 1A**

Densitometry was carried out as described in the methods to quantify data obtained in western blots of five similar experiments. Selected pairwise significant differences are shown, ***p<0.001, **p<0.01, *p<0.05.

**Supplementary Figure 2. Densitometry of blots in main figure 1B**

Densitometry was carried out as described in the methods to quantify data obtained in western blots in experiments comparing wild-type (WT) and knockout (KO) genotype in ES cells. ***p<0.001, **p<0.01. N=3 for each genotype

**Supplementary Figure 3. Densitometry of blots in main figure 2**

Densitometry was carried out as described in the methods to quantify data obtained in western blots. Bars significantly different from the amino acid withdrawal treatment (column 2) are shown, ***p<0.001, **p<0.01, *p<0.05. N=3

**Supplementary Figure 4. Densitometry of blots in main figure 3F**

Densitometry was carried out as described in the methods to quantify data obtained in western blots. Bars significantly different from the amino acid withdrawal treatment (column 2) are shown, ***p<0.001. N=3

**Supplementary Figure 5. Densitometry of blots in main figure 5A**

Densitometry was carried out as described in the methods to quantify data obtained in western blots. Bars significantly different from the amino acid withdrawal treatment (column 2) are shown, ***p<0.001, **p<0.01, *p<0.05. N=4

**Supplementary Figure 6. Effect of metformin and leucine on signalling in hepatocytes lacking AMPK** *(A-B)* Wild-type (WT) and AMPK double knockout (AMPK KO) hepatocytes were treated as in main Fig 1A. N=3

**Supplementary Figure 7. Interplay of leucine and metformin on colony formation** *(A,B)* Colony multiplication assays in soft agar were carried out as described in the Methods in the continued presence of the agents shown. After 3 weeks, the number of colonies was counted.

**Supplementary Figure 8. Gene expression responses of additional amino acid transporters**

*(A-C)* Cells were starved of amino acids for 3 hours in the presence or absence of metformin as shown and gene expression of LAT2 (A), LAT3 (B) and CAT2 (C) was measured by RTPCR as described in main Fig 4 and the Methods. (*D)* Hepatocytes lacking AMPK catalytic subunits were starved of amino acids for 3 hours in the presence or absence of metformin as shown and gene expression of SNAT2 was measured by RTPCR as described in the Methods. Each experiment was performed three times

**Supplementary Figure 9. Further plasma amino acid measurements** *(A-G)* Plasma was obtained from non-diabetic aged humans before and after 4 months of treatment with metformin (n=23, 2 g/day) or placebo (n=15) as described previously[^34^](#_ENREF_34). Plasma amino acid levels were determined as described in the Methods. Paired T-test analysis was carried out to determine differences before/after for arginine (A), cysteine (B), histidine (C), lysine (D), methionine (E), phenylalanine (F) and tryptophan (G). * denotes p<0.05 between first and second sample.

**Supplementary Figures**

**Fig. 1A pp70S6K**

**
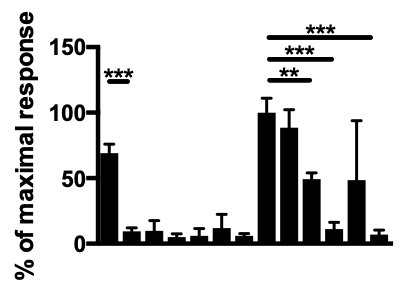
**

- + + + + + + + + + + + + Amino acid withdrawal

- - - - - - - - - - - + + Amino acid refeed

- - 1 2 5 - - - 1 2 5 - - Metformin (mM)

- - - - - 0.25 0.5 - - - - 0.25 0.5 Phenformin (mM)

- - 0.4 1 4 - 0.4 1 4 - 0.4 1 4 - 0.4 1 4 Leucine (mM)

- - - - - + + + + - - - - + + + + Metformin (2mM)

- - - - - - - - - + + + + + + + + Rapamycin (100nM)

**Fig 1B**

**pS6**

- + + + + + + + + + + + + Amino acid withdrawal

- - - - - - - - - - - + + Amino acid refeed

- - 1 2 5 - - - 1 2 5 - - Metformin (mM)

- - - - - 0.25 0.5 - - - - 0.25 0.5 Phenformin (mM)

- - 0.4 1 4 - 0.4 1 4 - 0.4 1 4 - 0.4 1 4 Leucine (mM)

- - - - - + + + + - - - - + + + + Metformin (2mM)

- - - - - - - - - + + + + + + + + Rapamycin (100nM)

**Fig 1C**

**pACC**

**
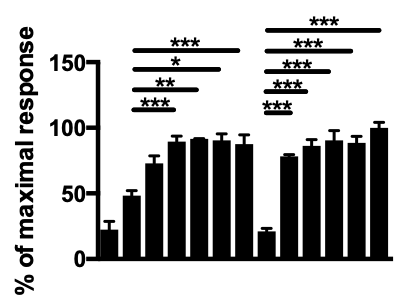
**

- + + + + + + + + + + + + Amino acid withdrawal

- - - - - - - - - - - + + Amino acid refeed

- - 1 2 5 - - - 1 2 5 - - Metformin (mM)

- - - - - 0.25 0.5 - - - - 0.25 0.5 Phenformin (mM)

- - 0.4 1 4 - 0.4 1 4 - 0.4 1 4 - 0.4 1 4 Leucine (mM)

- - - - - + + + + - - - - + + + + Metformin (2mM)

- - - - - - - - - + + + + + + + + Rapamycin (100nM)

**Fig 1D**

**pAMPK**

- + + + + + + + + + + + + Amino acid withdrawal

- - - - - - - - - - - + + Amino acid refeed

- - 1 2 5 - - - 1 2 5 - - Metformin (mM)

- - - - - 0.25 0.5 - - - - 0.25 0.5 Phenformin (mM)

- - 0.4 1 4 - 0.4 1 4 - 0.4 1 4 - 0.4 1 4 Leucine (mM)

- - - - - + + + + - - - - + + + + Metformin (2mM)

- - - - - - - - - + + + + + + + + Rapamycin (100nM)

**Fig 2A**

**pp70S6K**

**
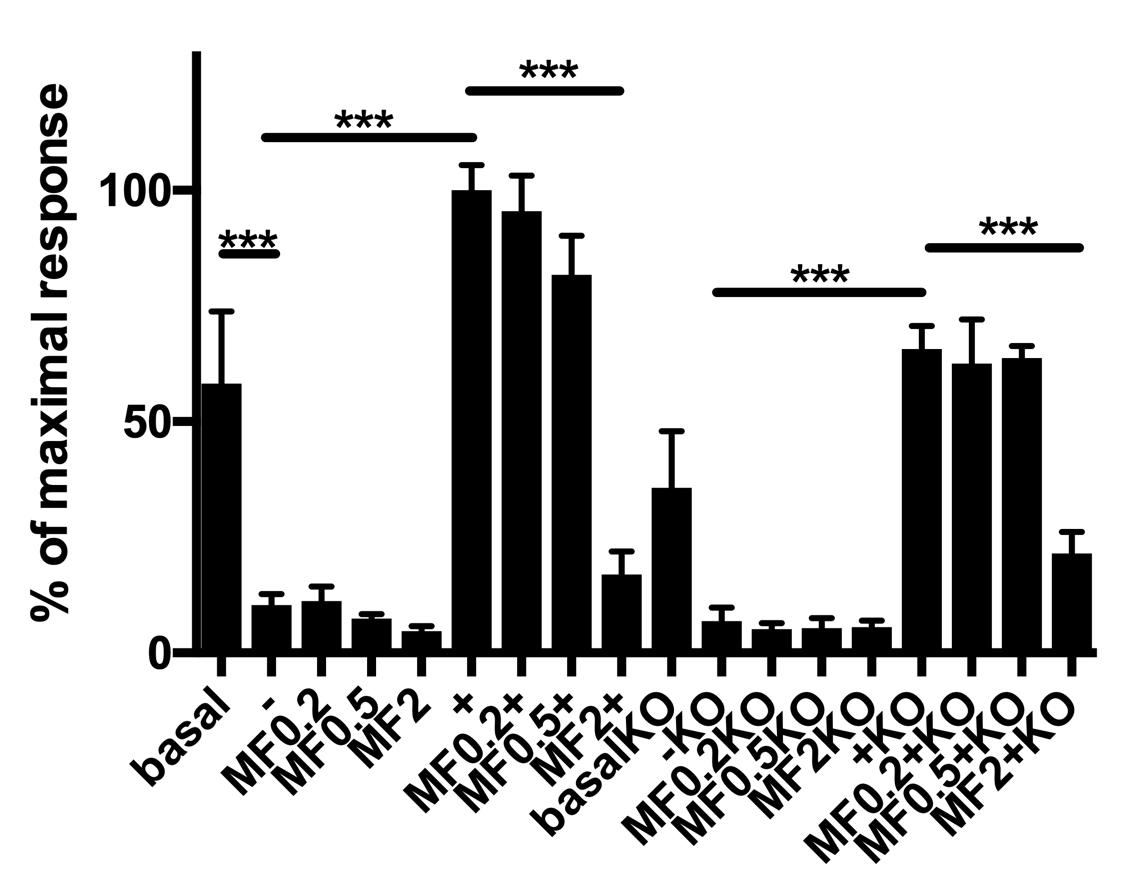
**

- + + + + + + + + - + + + + + + + + Amino acid withdrawal

- - - - - + + + + - - - - - + + + + Amino acid refeed

- - 0.25 0.5 2 - 0.25 0.5 2 - - 0.25 0.5 2 - 0.25 0.5 2 Metformin (mM)

WT KO

**Fig 2B pS6**

**
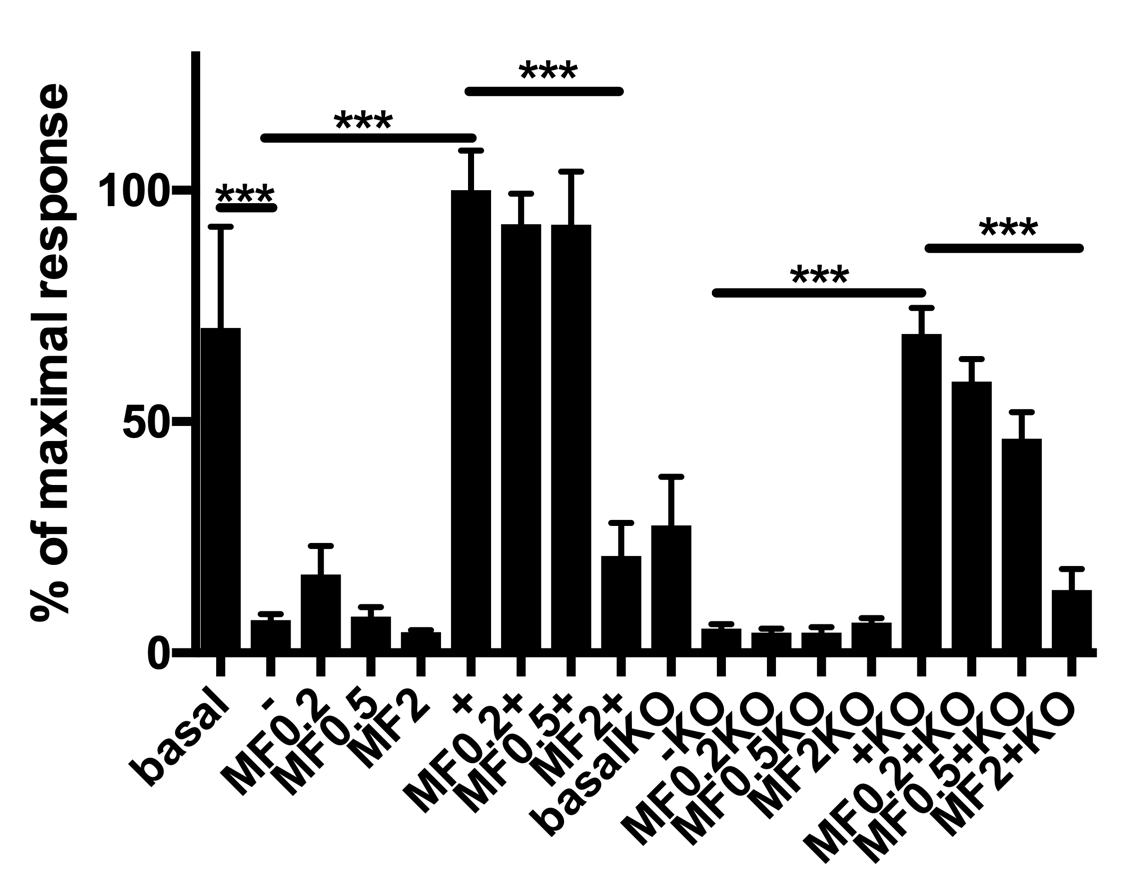
**

- + + + + + + + + - + + + + + + + + Amino acid withdrawal

- - - - - + + + + - - - - - + + + + Amino acid refeed

- - 0.25 0.5 2 - 0.25 0.5 2 - - 0.25 0.5 2 - 0.25 0.5 2 Metformin (mM)

WT KO

**Figure 2C pACC**

**
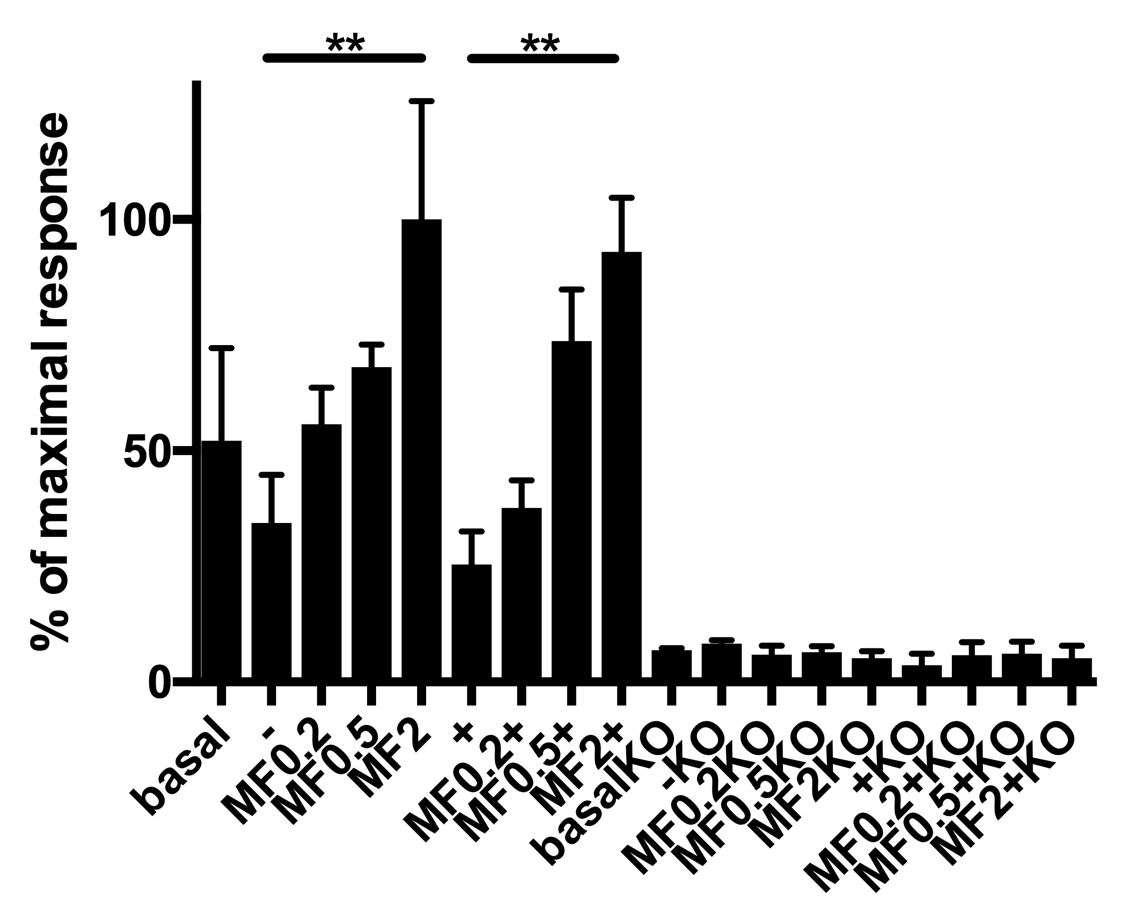
**

- + + + + + + + + - + + + + + + + + Amino acid withdrawal

- - - - - + + + + - - - - - + + + + Amino acid refeed

- - 0.25 0.5 2 - 0.25 0.5 2 - - 0.25 0.5 2 - 0.25 0.5 2 Metformin (mM)

WT KO

**Fig 2D pAMPK**

**
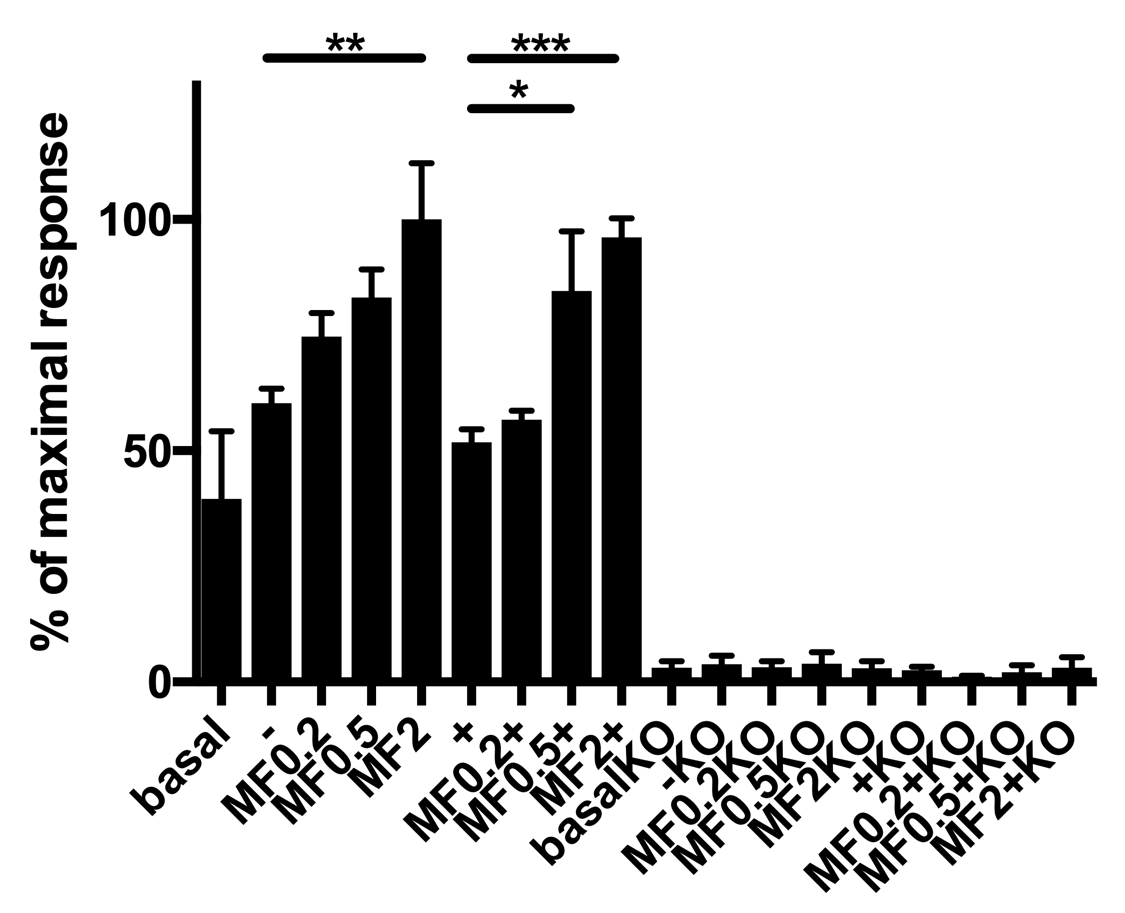
**

- + + + + + + + + - + + + + + + + + Amino acid withdrawal

- - - - - + + + + - - - - - + + + + Amino acid refeed

- - 0.25 0.5 2 - 0.25 0.5 2 - - 0.25 0.5 2 - 0.25 0.5 2 Metformin (mM)

WT KO

**Fig 3A pp70S6K**


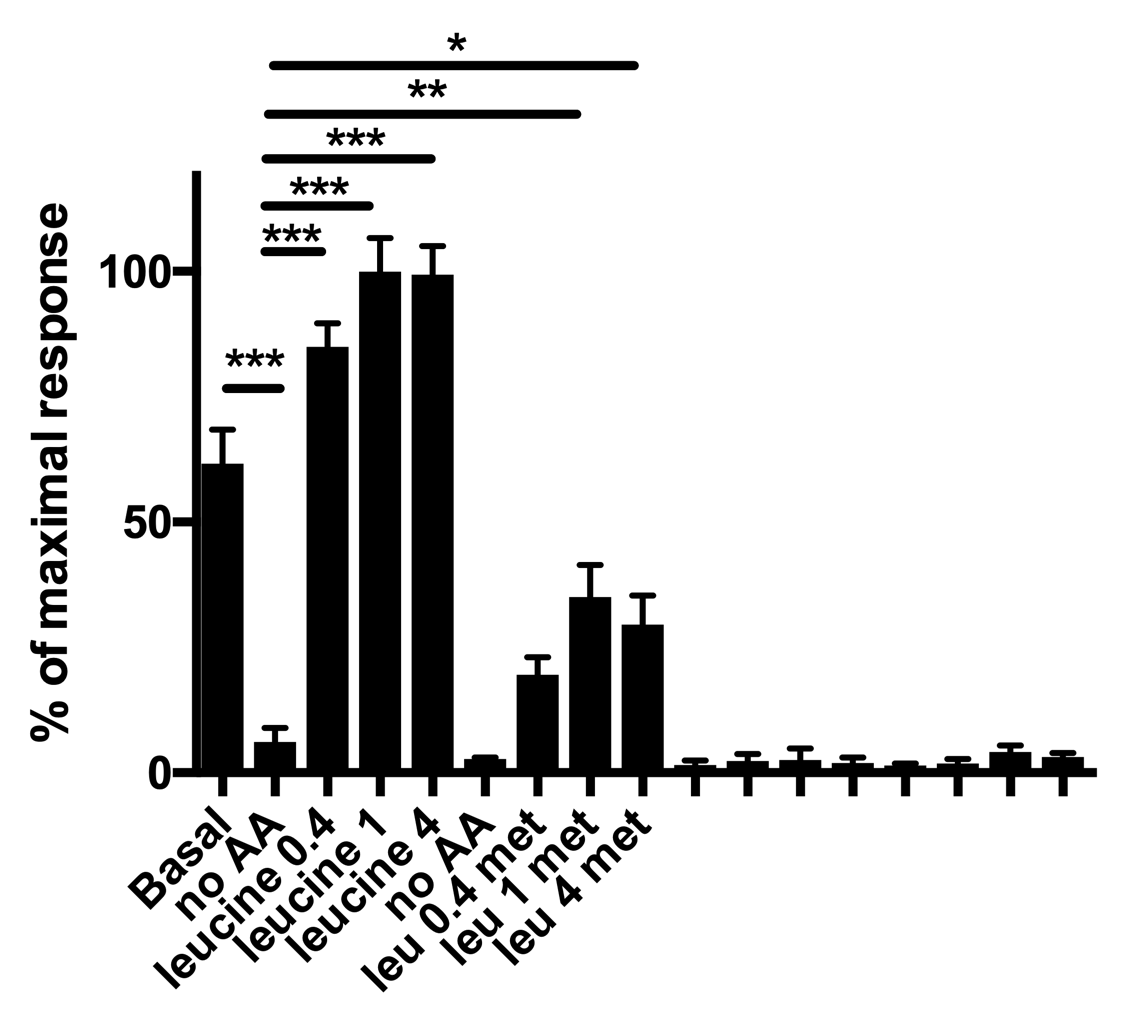


- + + + + + + + + + + + + + + + + Amino acid withdrawal

- - 0.4 1 4 - 0.4 1 4 - 0.4 1 4 - 0.4 1 4 Leucine (mM)

- - - - - + + + + - - - - + + + + Metformin (2mM)

- - - - - - - - - + + + + + + + + Rapamycin (100nM)


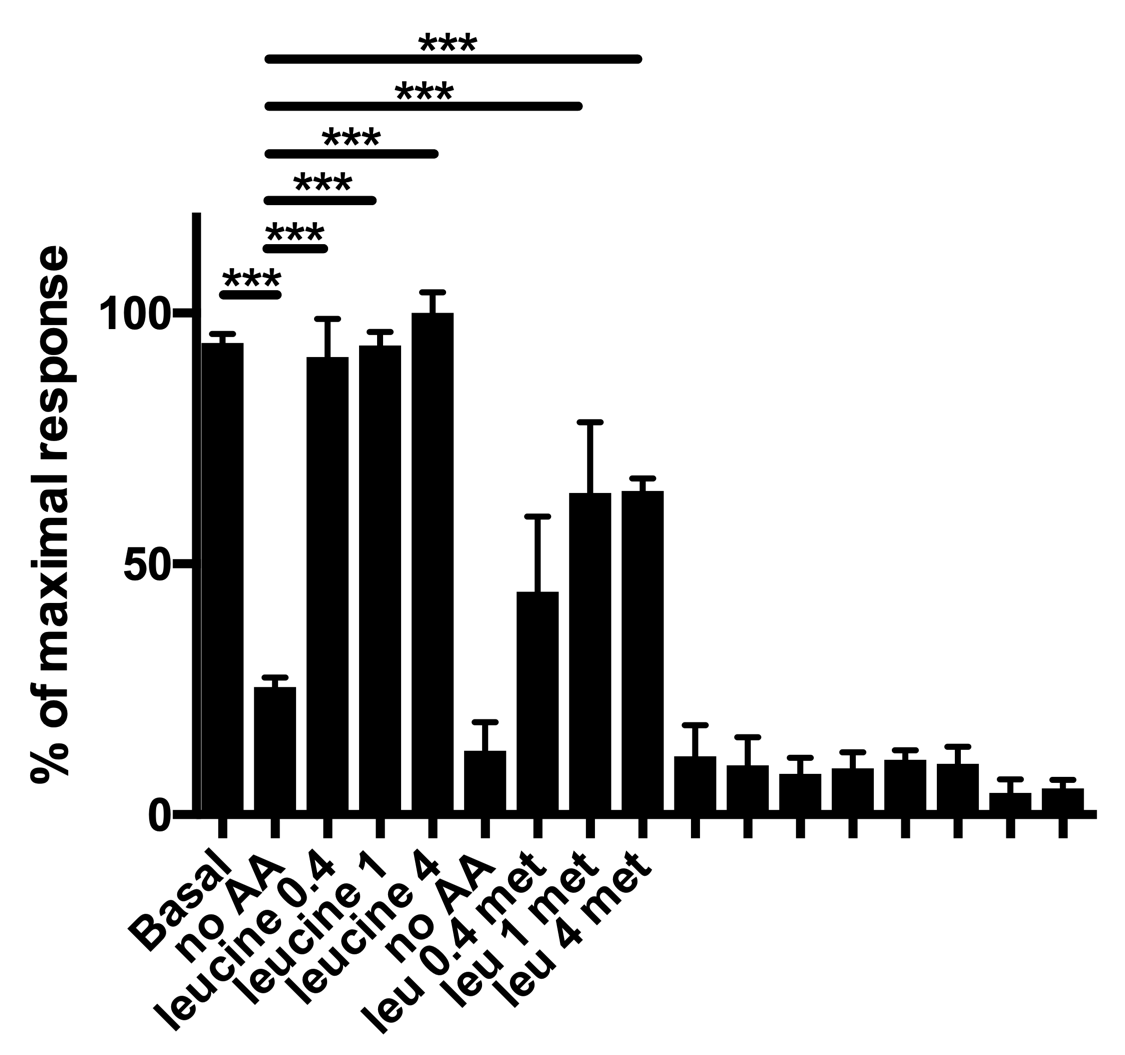


**Fig 3B**

**pS6**

- + + + + + + + + + + + + + + + + Amino acid withdrawal

- - 0.4 1 4 - 0.4 1 4 - 0.4 1 4 - 0.4 1 4 Leucine (mM)

- - - - - + + + + - - - - + + + + Metformin (2mM)

- - - - - - - - - + + + + + + + + Rapamycin (100nM)

**Fig 3C**

**pACC**


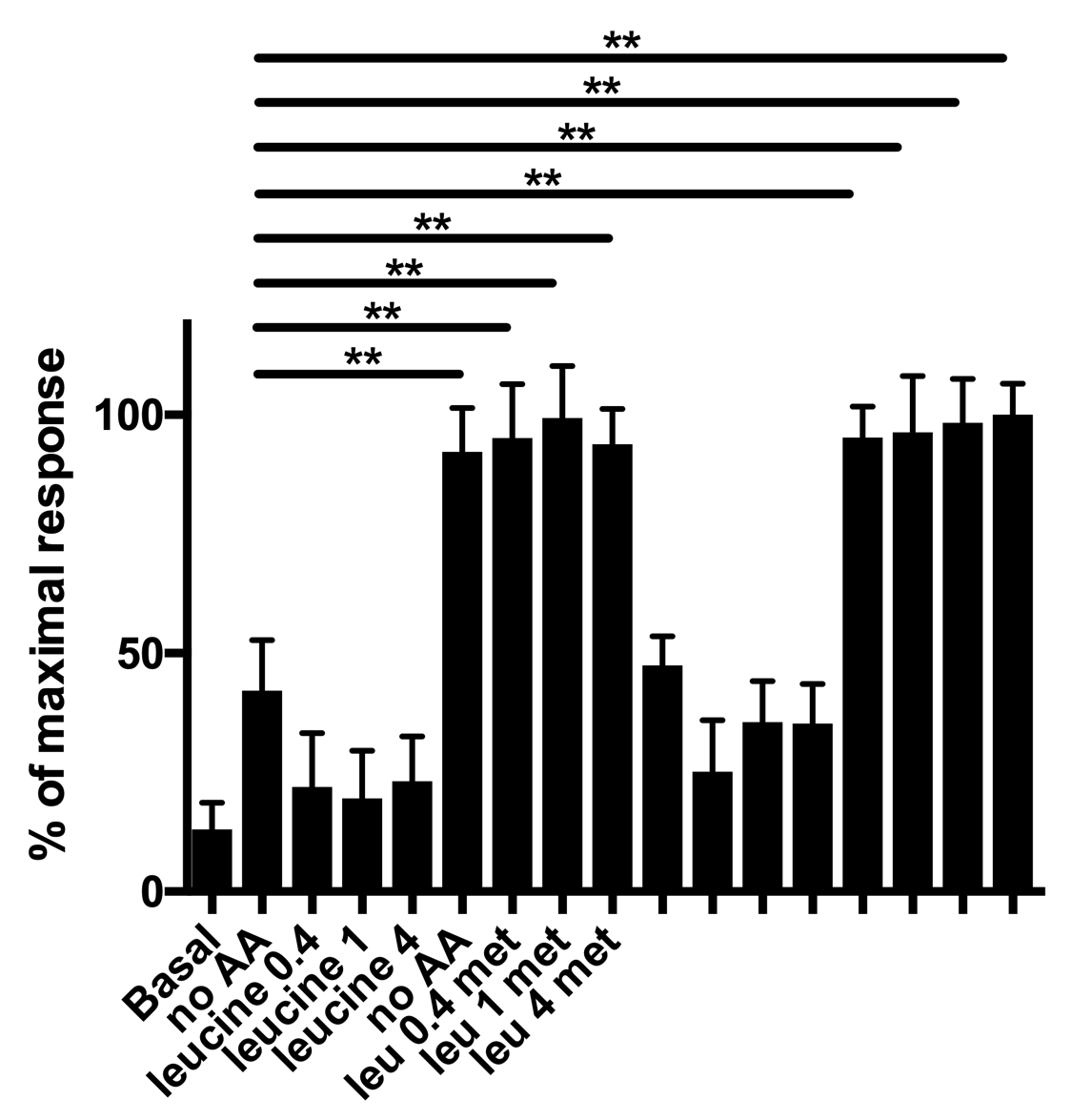


- + + + + + + + + + + + + + + + + Amino acid withdrawal

- - 0.4 1 4 - 0.4 1 4 - 0.4 1 4 - 0.4 1 4 Leucine (mM)

- - - - - + + + + - - - - + + + + Metformin (2mM)

- - - - - - - - - + + + + + + + + Rapamycin (100nM)


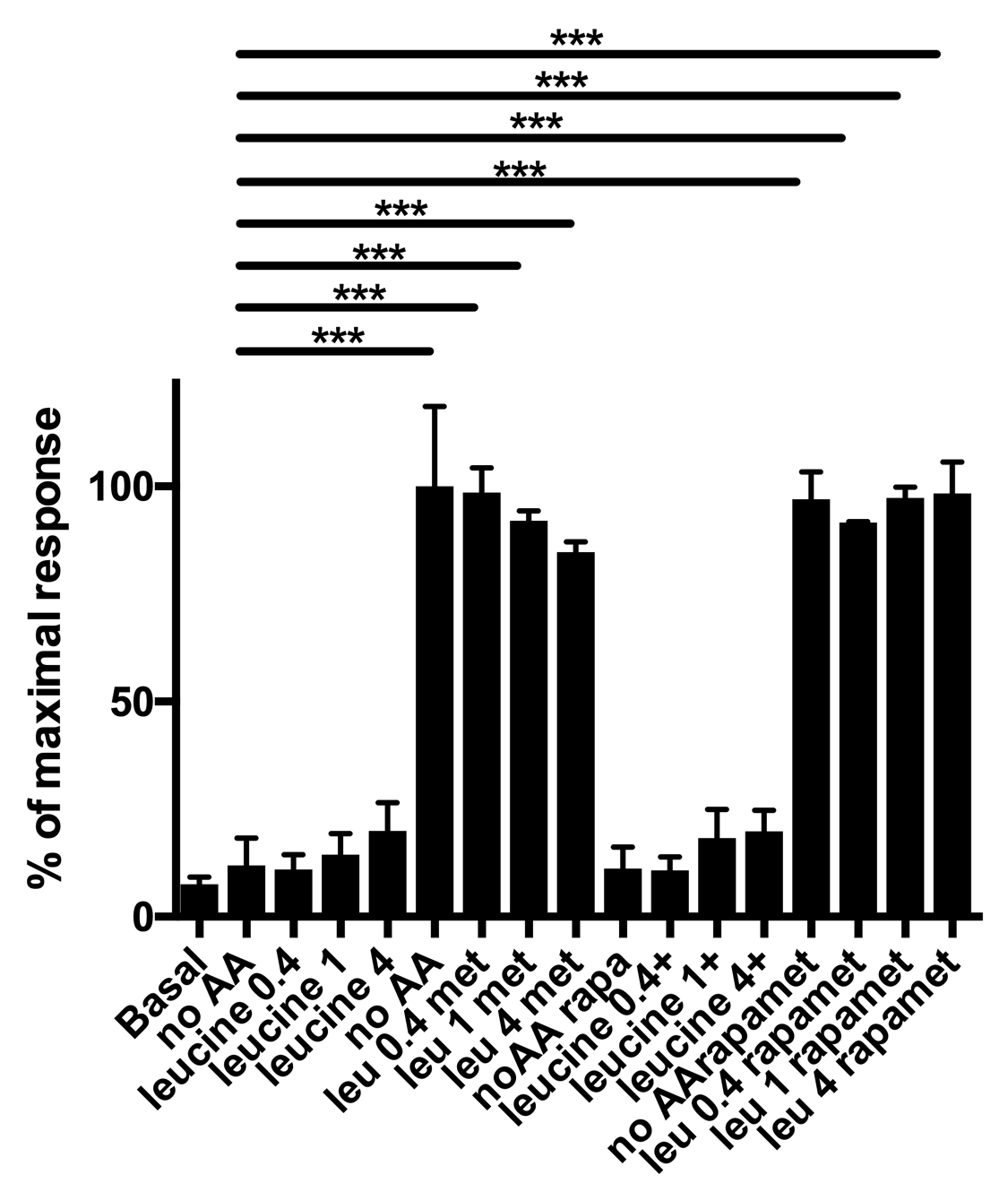


- + + + + + + + + + + + + + + + + Amino acid withdrawal

- - 0.4 1 4 - 0.4 1 4 - 0.4 1 4 - 0.4 1 4 Leucine (mM)

- - - - - + + + + - - - - + + + + Metformin (2mM)

- - - - - - - - - + + + + + + + + Rapamycin (100nM)

**Fig 3D**

**pAMPK**

**Fig 4 pp70S6K**

**
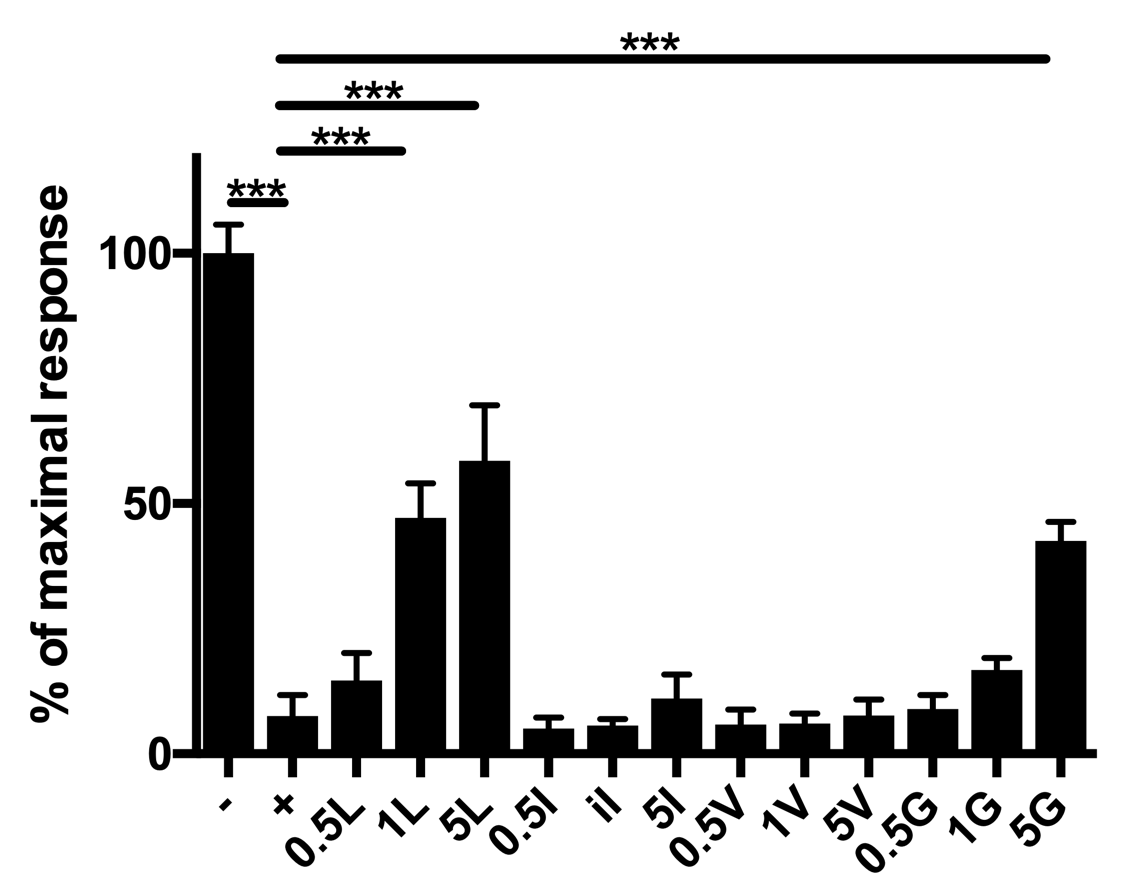
**

- + + + + + + + + + + + + + Amino acid withdrawal

- - 0.5 1 5 - - - - - - - - - Leucine (mM)

- - - - - 0.5 1 5 - - - - - - Isoleucine (mM)

- - - - - - - - 0.5 1 5 - - - Valine (mM)

- - - - - - - - - - - 0.5 1 5 Glutamine (mM)

**Fig 5 A pp70S6K**

- + + + + + + Amino acid withdrawal

- - - - + + + Amino acid refeed

- - 0.25 2 + 0.25 2 Metformin (mM)

**B pS6**

- + + + + + + Amino acid withdrawal

- - - - + + + Amino acid refeed

- - 0.25 2 + 0.25 2 Metformin (mM)

**Fig 5C pACC**

- + + + + + + Amino acid withdrawal

- - - - + + + Amino acid refeed

- - 0.25 2 + 0.25 2 Metformin (mM)

**D pAMPK**

- + + + + + + Amino acid withdrawal

- - - - + + + Amino acid refeed

- - 0.25 2 + 0.25 2 Metformin (mM)

**Supplementary Fig. 6**

**A**

**WT KO**

**WT KO**

**Supplementary Fig 7A**


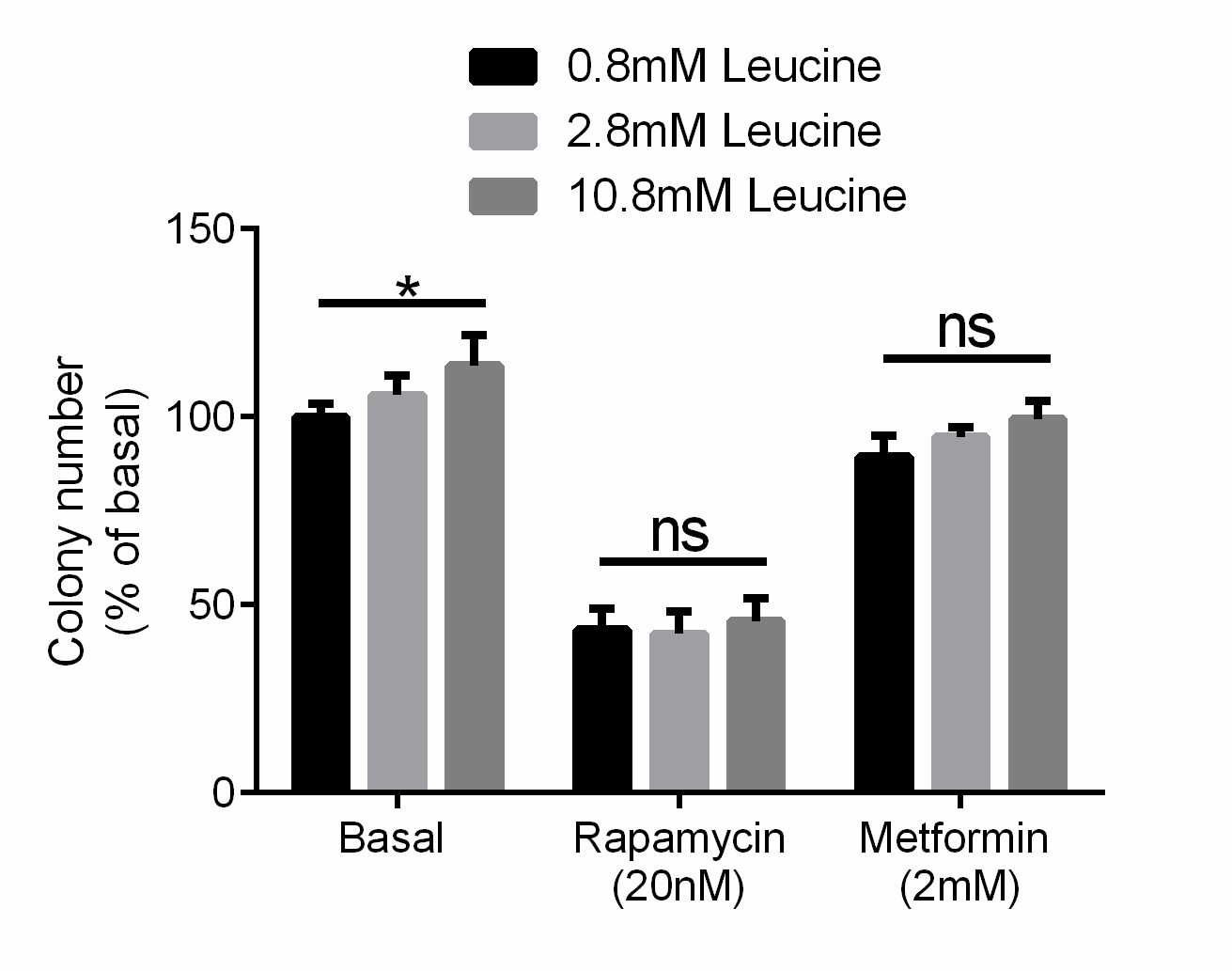


**B** Leucine

0.8mM 2.8mM 10.8mM


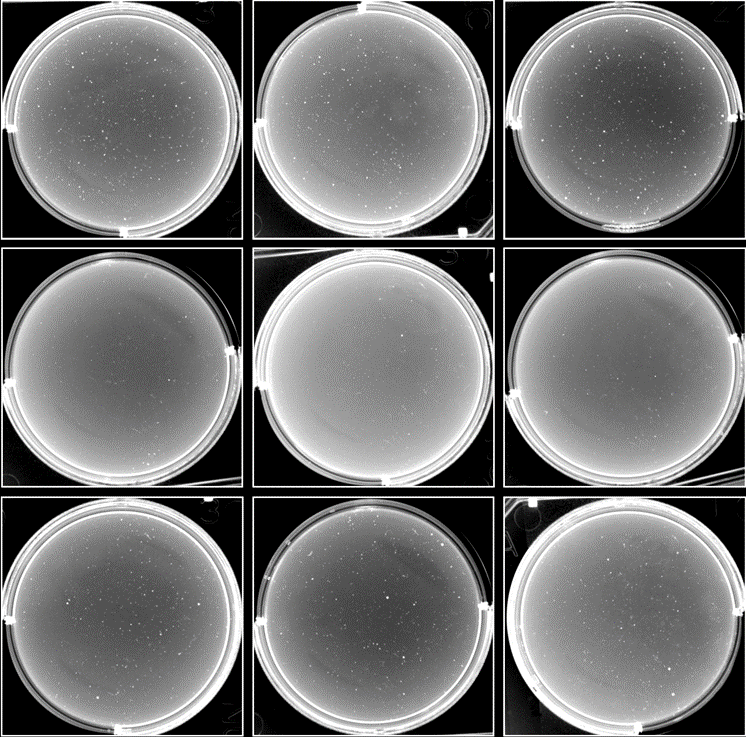


Basal

Rapamycin

(20nM)

Metformin

(2 mM)

**Fig 8 A**





**- + + + Amino acid withdrawal**

**0 0 0.1 2 Metformin (mM)**

**B**

**

**

**- + + + Amino acid withdrawal**

**0 0 0.1 2 Metformin (mM)**

**C**





**- + + + Amino acid withdrawal**

**0 0 0.1 2 Metformin (mM)**

**D**

**

**

**Figure 9A**

**



**

**B**

**

**

**

**

**C**

**



**

**D**

**



**

**E**

**



**

**F**

**



**

**G**

**



**
